# Geospatial joint modeling of vector and parasite serology to microstratify malaria transmission

**DOI:** 10.1073/pnas.2320898121

**Published:** 2024-06-04

**Authors:** Ellen A. Kearney, Punam Amratia, Su Yun Kang, Paul A. Agius, Kefyalew Addis Alene, Katherine O’Flaherty, Win Han Oo, Julia C. Cutts, Win Htike, Daniela Da Silva Goncalves, Zahra Razook, Alyssa E. Barry, Damien Drew, Aung Thi, Kyaw Zayar Aung, Htin Kyaw Thu, Myat Mon Thein, Nyi Nyi Zaw, Wai Yan Min Htay, Aung Paing Soe, James G. Beeson, Julie A. Simpson, Peter W. Gething, Ewan Cameron, Freya J. I. Fowkes

**Affiliations:** ^a^Disease Elimination Program, Burnet Institute, Melbourne, VIC 3004, Australia; ^b^Centre for Epidemiology and Biostatistics, Melbourne School of Population and Global Health, The University of Melbourne, Melbourne, VIC 3010, Australia; ^c^Malaria Atlas Project, Telethon Kids Institute, Perth, WA 6009, Australia; ^d^Biostatistics Unit, Faculty of Health, Deakin University, Melbourne, VIC 3125, Australia; ^e^Faculty of Health Sciences, Curtin University, Perth, WA 6102, Australia; ^f^Health Security and Malaria Program, Burnet Institute Myanmar, Yangon 11201, Myanmar; ^g^Department of Medicine at the Doherty Institute, The University of Melbourne, Melbourne, VIC 3000, Australia; ^h^Institute for Physical and Mental Health and Clinical Translation, School of Medicine, Deakin University, Geelong, VIC 3216, Australia; ^i^Department of Public Health, Myanmar Ministry of Health and Sports, Nay Pyi Taw 15011, Myanmar; ^j^Department of Infectious Diseases, The University of Melbourne, Melbourne, VIC 3000, Australia; ^k^Department of Microbiology, Monash University, Melbourne, VIC 3800, Australia; ^l^Central Clinical School, Monash University, Melbourne, VIC 3004, Australia; ^m^Department of Epidemiology and Preventive Medicine, Monash University, Melbourne, VIC 3004, Australia

**Keywords:** *Anopheles* salivary antibodies, malaria, geospatial, disease mapping

## Abstract

The World Health Organization identifies a strong surveillance system for malaria and its mosquito vector as an essential pillar of the malaria elimination agenda. *Anopheles* salivary antibodies are emerging biomarkers of exposure to mosquito bites that potentially overcome sensitivity and logistical constraints of traditional entomological surveys. Using samples collected by a village health volunteer network in 104 villages in Southeast Myanmar during routine surveillance, the present study employs a Bayesian geostatistical modeling framework, incorporating climatic and environmental variables together with *Anopheles* salivary antigen serology, to generate spatially continuous predictive maps of *Anopheles* biting exposure. Our maps quantify fine-scale spatial and temporal heterogeneity in *Anopheles* salivary antibody seroprevalence (ranging from 9 to 99%) that serves as a proxy of exposure to *Anopheles* bites and advances current static maps of only *Anopheles* occurrence. We also developed an innovative framework to perform surveillance of malaria transmission. By incorporating antibodies against the vector and the transmissible form of malaria (sporozoite) in a joint Bayesian geostatistical model, we predict several foci of ongoing transmission. In our study, we demonstrate that antibodies specific for *Anopheles* salivary and sporozoite antigens are a logistically feasible metric with which to quantify and characterize heterogeneity in exposure to vector bites and malaria transmission. These approaches could readily be scaled up into existing village health volunteer surveillance networks to identify foci of residual malaria transmission, which could be targeted with supplementary interventions to accelerate progress toward elimination.

## Significance

Evaluation of human antibodies against *Anopheles* salivary proteins has emerged as a sensitive and feasible advancement on traditional entomological methods to quantify exposure to vector bites and malaria transmission. Using samples collected during routine malaria testing by village health volunteers, our study inputs serological biomarkers of vector and parasite exposure into a geospatial modeling framework to generate fine-scale predictive maps of *Anopheles* biting exposure and malaria transmission intensity. Our predictions advance current maps of only vector occurrence, and our methodology suggests a framework that could be readily expanded into a surveillance platform to identify high-risk areas for targeted intervention delivery planning.

The rapid spread of resistance to frontline antimalarial drugs throughout the Greater Mekong Subregion (GMS) has seen the countries of the region agree to accelerate efforts toward elimination with the aim to declare the GMS malaria-free by 2030. Since then, the countries of the GMS have significantly reduced the incidence of malaria (1). As malaria transmission declines in the GMS, it becomes increasingly heterogeneous, localizing in discrete geographical foci and high-risk populations (2). A strong surveillance system for malaria and its vector, the *Anopheles* mosquito, to capture this heterogeneity has been identified by the World Health Organization as a pillar of the GMS malaria elimination agenda to ensure that appropriate and effective interventions are targeted to areas with the greatest need to accelerate transmission decline, and use limited resources effectively (3). However, due to logistical constraints, there is a dearth of entomological surveys that collect vector endpoints that are used to microstratify vector exposure and malaria transmission risk. New logistically feasible tools are required to measure fine-scale exposure to vector bites and model the consequent geospatial microheterogeneity in malaria transmission to inform intervention delivery.

The detection of human antibody biomarkers against *Anopheles* salivary proteins is an emerging approach to measure exposure to vector bites. This individual-level data-rich approach advances on entomological surveys which provide a crude measure of vector density at a chosen collection site and are difficult to conduct at scale. A recent systematic review and meta-analysis provides important evidence of the positive association between antibodies to the *Anopheles* salivary gland 6 (SG6) antigen and human biting rate (HBR: the number of bites received per person per unit of time) (4). This suggests that SG6 antibodies could serve as a sensitive and feasible proxy measure for HBR, potentially overcoming the logistical challenges of the gold standard method, the human landing catch, where participants directly capture mosquitoes that land on an exposed limb. The systematic review also showed that while anti-SG6 antibodies were associated with the gold standard measure of malaria transmission intensity [the entomological inoculation rate (EIR): the number of infective bites received per person per unit of time (5, 6)], the association was not as strong as with HBR (4). This suggests that anti-SG6 antibodies (serving as a proxy for HBR) would likely need to be combined with an additional metric representative of the sporozoite index (proportion of infected mosquitos) to provide an accurate proxy measure of EIR. We hypothesize that antibodies specific for the circumsporozoite (CSP) protein expressed on the transmitting sporozoite stage of the *Plasmodium* spp. parasite which causes malaria, and serves as a marker of recent exposure (and not just current infection) (7, 8), could be useful in this context. Combining anti-SG6 and CSP antibody metrics may provide a feasible approach to estimate recent vector and parasite exposure at the individual level and help to overcome logistical constraints, sampling bias, and sensitivity limitations of EIR estimates in settings approaching elimination due to difficulties in capturing the few parasite-positive mosquitoes (9).

Using an ecological geospatial modeling framework, the present study explores the use of *Anopheles* salivary biomarkers as a metric with which to microstratify malaria transmission risk. Validated using samples collected by village health volunteers during routine malaria testing in Southeast Myanmar, our study aims are threefold. First, we aimed to use a geostatistical modeling approach to identify climatic and environmental covariates associated with SG6 IgG seroprevalence (as a biomarker for HBR) and quantify and characterize the spatial heterogeneity observed. Second, using these associations, we aimed to predict spatially continuous estimates and investigate seasonal patterns of SG6 IgG seroprevalence across three states in Southeast Myanmar. Third, we aimed to explore combining antibodies against SG6 and CSP (as biomarkers of exposure to *Anopheles* bites and transmission stage parasites) in a serological joint geospatial model, as an innovative approach to measure malaria transmission.

## Results

### Prevalence of Malaria and Serological Biomarkers.

A total of 13,594 samples collected by village health volunteers from 104 villages across Bago (East), Kayah and Kayin states in Southeast Myanmar between April 2015 and June 2016 were available for use in this analysis (*SI Appendix*, Table S1 and Fig. S18). Overall, the median age of participants was 19 y [interquartile range (IQR): 10 to 35] and 49.5% (6,723/13,594) were male. The majority of samples came from forest-goers (i.e., regular work and/or overnight stay in forested areas) (46.8%; 6,364/13,594) and village residents (42.6%; 5,795/13,594), compared to migrants (10.5%; 1,433/13,594). A total of 404 *Plasmodium* spp. infections were detected by PCR (3.2%; 404/12,678); 198 *P. falciparum* infections (1.56%), 120 *P. vivax* infections (0.95%); and 86 mixed infections (0.68%). The overall seroprevalence of anti-SG6 IgG was 59.4% (8,077/13,594), while antibodies against *P. falciparum* and *P. vivax* sporozoite antigens were similar [*Pf*CSP 18.5% (2,520/13,594), *Pv*CSP 18.6% (2,298/12,363)].

**Fig. 1.**
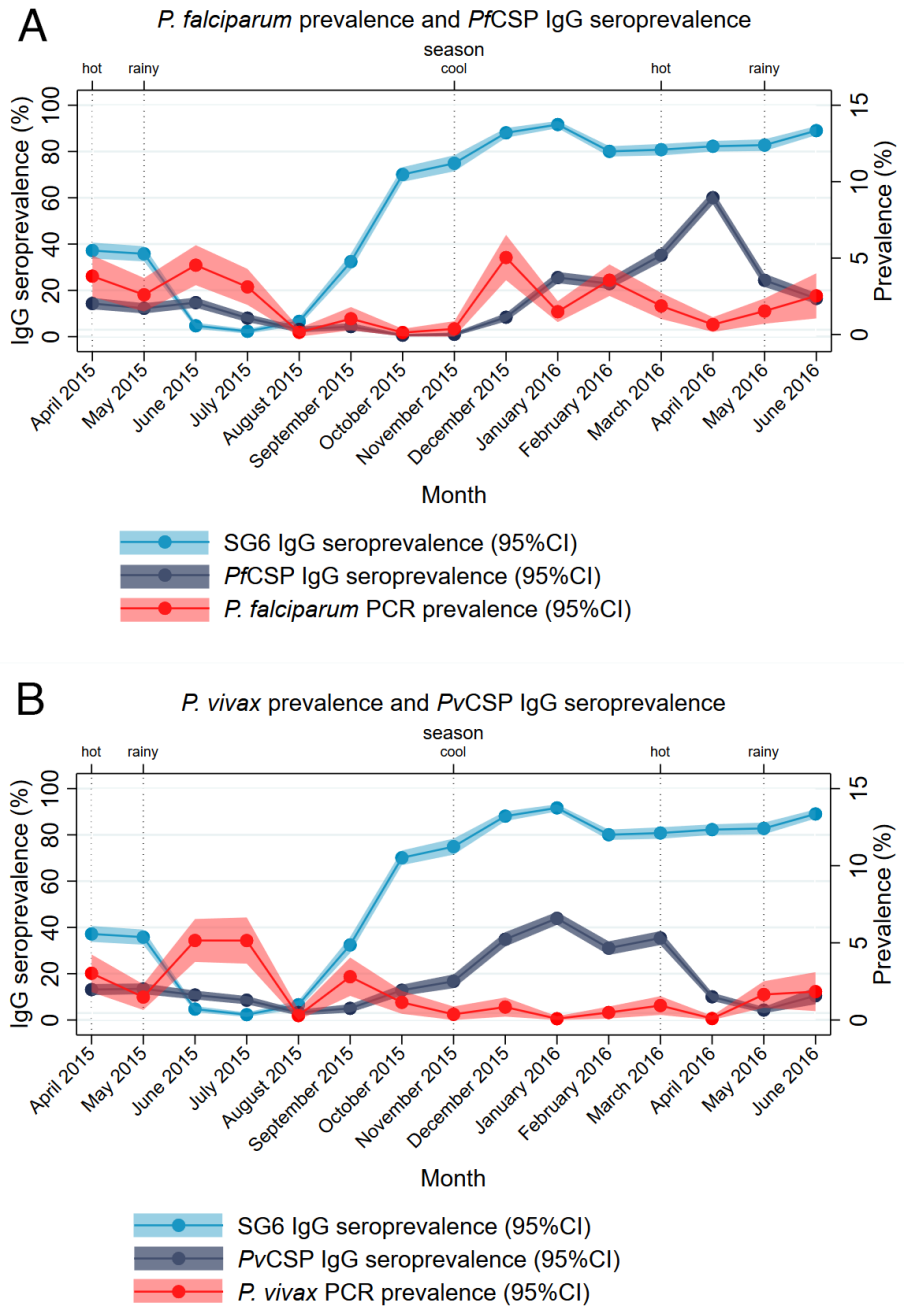
Overall anti-SG6 IgG seroprevalence, with (*A*) *P. falciparum* and (*B*) *P. vivax* prevalence and anti-CSP IgG over time. Fig. 1*A* shows the seroprevalence and 95% CI of IgG to *P. falciparum* transmission stage (*Pf*CSP) and the vector salivary (SG6) antigens (*Left* *y* axis), as well as the prevalence (95% CI) of *P. falciparum* infection (*Right* *y* axis), over the 15-mo study period. Fig. 1*B* shows the seroprevalence (95% CI) of IgG to *P. vivax* transmission stage (*Pv*CSP) and the vector salivary (SG6) antigens (*Left* *y* axis), as well as the prevalence (95% CI) of *P. vivax* infection (*Right* *y* axis), over the 15-mo study period. Vertical dotted lines indicate typical season.

The seroprevalence and levels of anti-SG6 IgG antibodies changed over time (Fig. 1 and *SI Appendix*, Fig. S1 respectively), highlighting seasonal patterns. Monthly rainfall and day-time land surface temperatures are presented in *SI Appendix*, Fig. S2. We observed a decline in anti-SG6 IgG seroprevalence from 35 to 5%, coinciding with the end of the hot and beginning of the rainy season (April to June 2015). This very low seroprevalence (2 to 7%) was maintained for the first 3 mo of the rainy season before increasing throughout the remainder of the rainy season and into the cool, reaching a peak of 92% in January 2016 and remaining higher than 80% until the end of the study in June 2016. Similar temporal patterns were observed for PCR-detectable *Plasmodium* spp. prevalence and antibodies to *P. falciparum* and *P. vivax* sporozoite CSP antigens (see Fig. 1) with the highest seroprevalence observed in the cool and hot seasons of 2015 and 2016. Peaks of anti-*Pf*CSP, but not *Pv*CSP IgG, coincided with respective peaks of *P. falciparum* and *P. vivax* infections in the cool season of 2015/2016.

### Associations between Anti-SG6 IgG Seroprevalence and Vectorial, Climatic, and Environmental Covariates.

In order to understand drivers of *Anopheles* exposure, we first sought to determine the univariate associations between anti-SG6 IgG seroprevalence and our vectorial, climatic, and environmental covariates that were identified a priori as being associated with vector occurrence (Table 1). Each covariate [comprising a satellite-derived raster of Bago (East), Kayah and Kayin states in Southeast Myanmar] was included in turn in a univariate Bayesian geostatistical model. Briefly, we identified mostly positive associations between anti-SG6 IgG seroprevalence and climatic variables, i.e., sum annual rainfall [odds ratio (OR): 1.40, 95% credible interval (CrI): 1.12, 1.76], potential evapotranspiration (OR: 1.25, 95%CrI: 1.04, 1.50) and the day (OR: 1.23, 95%CrI: 1.03, 1.48), night (OR: 1.14, 95%CrI: 1.01, 1.29), and diurnal difference (OR: 1.05, 95%CrI: 0.90, 1.23) land surface temperatures. Environmental variables showed a negative association between anti-SG6 IgG seroprevalence and the enhanced vegetation index (OR: 0.89, 95%CrI: 0.76, 1.05) and tree coverage fraction (OR: 0.68, 95%CrI: 0.56, 0.83), as well as elevation (OR: 0.94, 95%CrI: 0.82, 1.09) and topographical slope (OR: 0.96, 95%CrI: 0.89, 1.03). We observed positive associations between anti-SG6 IgG seroprevalence and population density (OR: 1.12, 95%CrI: 1.02, 1.24), night-time lights (OR: 1.14, 95%CrI: 1.04, 1.26), and tasseled cap brightness (OR: 1.14, 95%CrI: 1.03, 1.26), but a negative association with inaccessibility to cities (OR: 0.84, 95%CrI: 0.75, 0.94).

**Table 1.** Geostatistical model outputs of anti-SG6 IgG seroprevalence and with each vectorial, climatic, and environmental covariate considered for inclusion

Variable	Description	OR	95%CrI	
Vectorial				
*An. dirus* occurrence (10) (probability)	MAP predicted occurrence	1.01	0.85	1.20
*An. minimus* occurrence (10) (probability)	MAP predicted occurrence	1.27	1.06	1.53
*An. maculatus* occurrence (10) (probability)	MAP predicted occurrence	0.79	0.33	1.89
Climatic				
Aridity index (11)		1.21	0.94	1.56
Potential evapotranspiration (11) (mm/t)		1.25	1.04	1.50
Rainfall (12) (mm)	2015 sum annual	1.40	1.12	1.76
Land surface temperature (day) (13) (°C)	2015 mean annual	1.23	1.03	1.48
Land surface temperature (night) (13) (°C)	2015 mean annual	1.14	1.01	1.29
Land surface temperature (diurnal difference) (13) (°C)	2015 mean annual (day–night difference)	1.05	0.90	1.23
Temperature Suitability Index for *P. falciparum* (14)		1.20	1.07	1.35
Environmental				
Distance to water (15) (meters)	Measure of distance to lakes, wetlands, rivers, and streams, accounting for slope and precipitation	1.21	0.99	1.48
Tasselled Cap Wetness Index (16)	Measure of wetness, i.e., soil moisture, water, etc.	0.84	0.75	0.94
Topographic Wetness Index (17)	Elevation derived	1.06	0.95	1.18
Elevation (17) (meters)		0.94	0.82	1.09
Slope (17)	Elevation derived	0.96	0.89	1.03
Enhanced Vegetation Index (18, 19)	2015 mean annual	0.89	0.76	1.05
Tree coverage fraction (% forest cover) (20)		0.68	0.56	0.83
Tasselled Cap Brightness Index (16)	Measure of land reflectance, i.e., manmade structures, barren/rocky ground	1.14	1.03	1.26
Inaccessibility (21) (minutes)	Travel time to cities with population <5,000	0.84	0.75	0.94
Population density (people/pixel) (22, 23)		1.12	1.02	1.24
Night-time lights (24)	Measures of presence of lights, i.e., cities, towns, etc.	1.14	1.04	1.26

Note. Data given as OR with 95%CrI for each (standardized) covariate fitted in univariate Bayesian spatial models of the binomial response for the seroprevalence of anti-SG6 IgG antibodies.

### Geospatial Maps of Anti-SG6 IgG Seroprevalence Show Fine-scale Spatial and Temporal Heterogeneity.

**Fig. 2.**
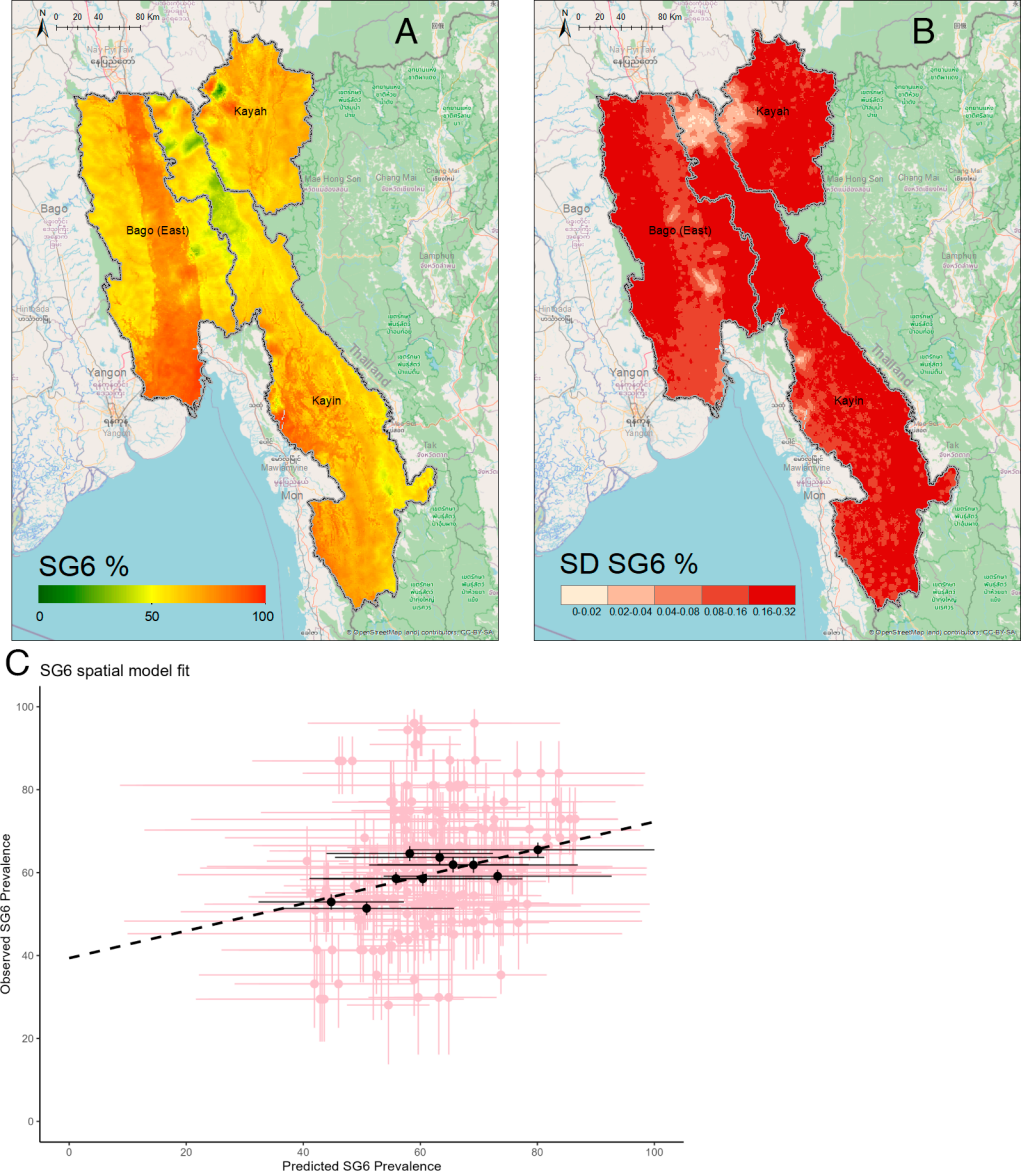
Predicted anti-SG6 IgG seroprevalence and model uncertainty for Bago (East), Kayah and Kayin, with model validation. Geospatial maps showing (*A*) the predicted posterior mean probability and (*B*) SD of anti-SG6 IgG seropositivity. Estimated using a geospatial model that adjusts for rainfall, distance to water, potential evapotranspiration, tree coverage, and diurnal temperature difference. Model validation (*C*): The model is trained on data from a random sample of 90% of villages (20 repeats), with internal validity assessed as the correlation between the observed vs. predicted SG6 seroprevalence (with 95%Crl) for each of the omitted 10% of villages (pink crosses) and for omitted villages grouped in a series of bins (deciles) by predicted seroprevalence (black dots). Pearson correlation used to estimate r = 0.731

After assessing for multicollinearity and undergoing stepwise model selection of environmental and climatic variables, a geostatistical model-based estimate for the seroprevalence of IgG antibodies against the *Anopheles* salivary antigen SG6 across Bago (East), Kayah and Kayin in Southeast Myanmar was determined as shown in Fig. 2. Fig. 2*A* shows the predicted posterior mean seroprevalence of anti-SG6 IgG at a 1 km × 1 km resolution (which ranged from 9 to 99%), while Fig. 2*B* shows the SD of the pixel-wise predicted probability as an indication of uncertainty in our model. Table 2 provides the estimated regression coefficients for the covariates included in the prediction model. We observed a weak positive association between anti-SG6 IgG seroprevalence and rainfall (OR: 1.27, 95%CrI: 0.91, 1.77), but a negative association with the diurnal temperature difference (OR: 0.81, 95%CrI: 0.65, 1.00). We also observed positive associations between anti-SG6 IgG seroprevalence and our environmental variables: distance to water (OR: 1.37, 95%CrI: 1.10, 1.71) and potential evapotranspiration (OR: 1.38, 95%CrI: 1.12, 1.70) and negative associations with tree coverage (OR: 0.59, 95%CrI: 0.43, 0.81). Fig. 2*C* shows the model validation which indicates good model fit and predictive accuracy (r = 0.731).

**Table 2.** Regression coefficient and 95%Crl of covariates fitted in our Bayesian spatial model of the binomial response for the seroprevalence of anti-SG6 IgG antibodies

Covariate	OR	95% CrI	
b0	2.06	1.41	3.02
Rainfall	1.27	0.91	1.77
Land surface temperature diurnal difference	0.81	0.65	1.00
Distance to water	1.37	1.10	1.71
Potential evapotranspiration	1.38	1.12	1.70
Tree coverage fraction	0.59	0.43	0.81

Note. Data given as OR and 95%CrI for a 1 SD change in each covariate fitted in a Bayesian geostatistical model of the binomial response for the seroprevalence of anti-SG6 IgG antibodies using all data (n = 13,594).

Here, we present probabilistic maps of the seroprevalence of antibodies against the *Anopheles* salivary protein SG6 as a proxy biomarker for exposure to *Anopheles* bites (Fig. 2). Overall, we show that the seroprevalence of anti-SG6 IgG was high (mean: 66%), but with fine-scale spatial heterogeneity (ranging from 9 to 99%) across the three states of interest. Large areas of Bago (East), the South-Western part of Kayin, and the North-Eastern part of Kayah all predict very high seroprevalence of anti-SG6 IgG antibodies (~75 to 95%). While the Northern section of Kayin contains clusters of lower seroprevalence (~25 to 35%) of anti-SG6 antibodies particularly along borders shared with both Kayah and Bago (East).

We also present maps of the anti-SG6 seroprevalence for the hot, rainy, and cool seasons (*SI Appendix*, Fig. S3). As low monthly sampling in numerous villages prevented the development of a spatiotemporal model; data were instead partitioned by season and separate geostatistical models were fitted to each stratified dataset (*SI Appendix*, Discussion 1). Interestingly, while the seroprevalence of anti-SG6 IgG antibodies is markedly different depending on seasonality, the patterns of spatial heterogeneity in anti-SG6 IgG seroprevalence are similar between each season and when considered altogether (*SI Appendix*, Fig. S3). Similarly, while the overall predicted distribution of anti-SG6 IgG seroprevalence was higher using samples collected in high-risk participants (migrants and forest-goers) compared to village residents, the spatial patterns and hot spots were similar (*SI Appendix*, Fig. S5).

### Serological Joint Modeling Framework to Identify Foci of Malaria Transmission.

In order to microstratify malaria risk and identify foci of malaria transmission, we developed a serological joint modeling framework combining antibody biomarkers of exposure to vector bites (*Anopheles* salivary SG6) and the transmission stage of the parasite (CSP) as a proxy measure of malaria transmission (EIR). Rather than modeling our three outcomes [SG6, CSP (seropositivity to either/both *Pf*CSP and *Pv*CSP), and PCR detectable *Plasmodium* spp. infections] as several univariate datasets, we assumed some level of relatedness between the outcomes and included them in a joint model with multiple likelihoods, as an innovative alternative approach to microstratify malaria transmission risk. This joint dependency structure is directly modeled as shared components at the predictor level. After undergoing a stepwise model selection procedure to identify the covariates of interest and estimate their associations with anti-SG6 IgG antibodies; the model then uses this to estimate the associations with anti-CSP IgG antibodies, and then, the two combined are used to estimate associations with PCR-detectable *Plasmodium* spp. infections (*SI Appendix*, Table S4).

**Fig. 3.**
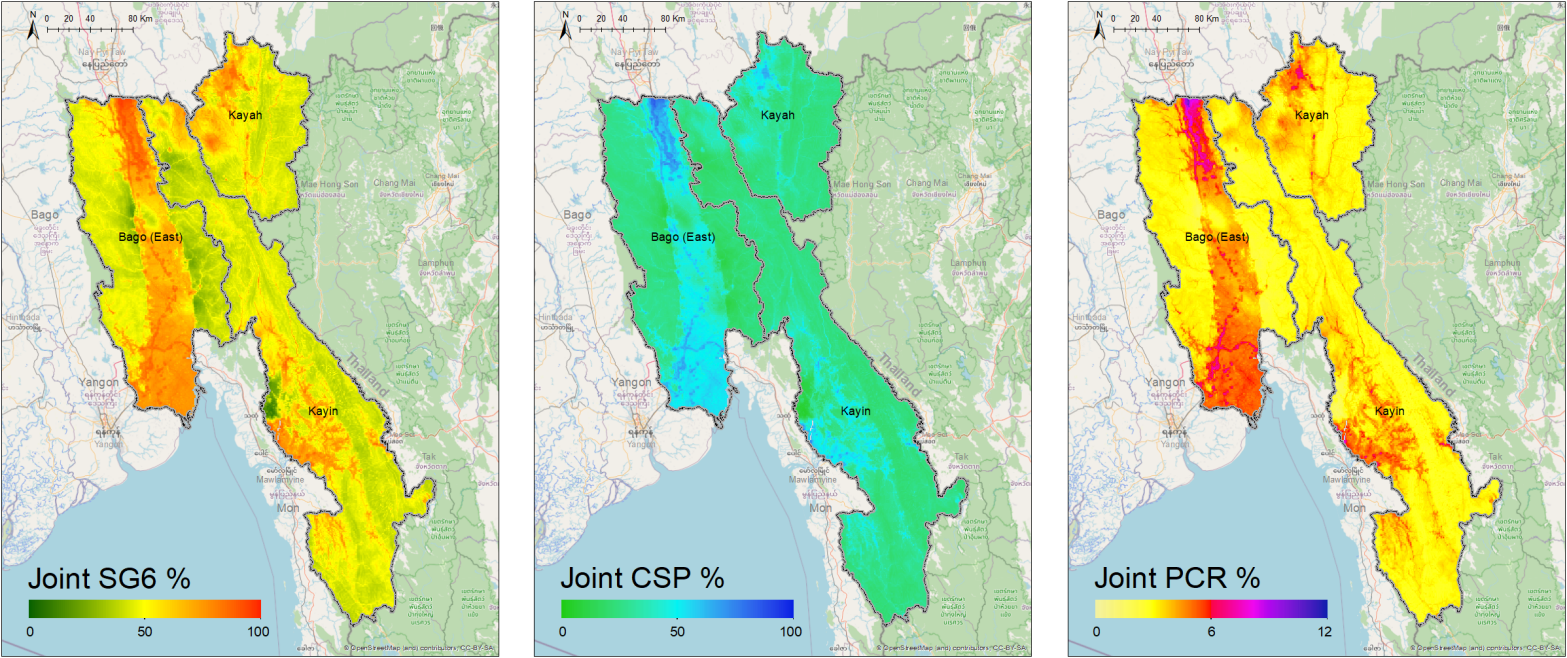
Predicted seroprevalence of anti-SG6 and CSP IgG antibodies and predicted prevalence of PCR-detectable *Plasmodium* spp. infections after joint modeling of these outcomes. Estimated using a geospatial model that adjusts for distance to water, topographical wetness index, slope, tree coverage fraction, inaccessibility to cities, and night-time lights (models were fitted to data from participants in all villages who had observations for all outcomes, n = 11,988).

Fig. 3 shows the predicted posterior seroprevalence of anti-SG6 and CSP IgG antibodies, as well as the predicted posterior prevalence of PCR-detectable infections from the joint modeling. We show that inclusion in the joint model strengthens the associations between our outcomes and our models have reasonable predictive power (*SI Appendix*, Discussion 3 and Figs. S7–S10).

The probabilistic maps generated by joint modeling of the three outcomes show fine-scale spatial heterogeneity in the seroprevalence of anti-SG6 (mean: 53%, range: 15 to 92%) and CSP IgG (mean: 30%, range: 8 to 74%), and prevalence of PCR detectable *Plasmodium* spp. infections (mean: 3%, range: 2 to 8%), across Bago (East), Kayah and Kayin. These maps predict several geographical foci with a high prevalence of *Plasmodium* spp. infections. Through the central part of Bago (East), notably following a road and train route running north–south, as well as along border areas—including along the Sittang River in the north where it borders Nay Pyi Taw (~5 to 8%) and to the south where it borders Yangon and Mon State (~4 to 5%). Similarly, in Kayin State, we observed higher prevalence of *Plasmodium* spp. infections in the southwest at the border with Mon State and along the Gyaing and Hliangbwe Rivers (~4 to 6%). Additional hot spots of *Plasmodium* spp. infection can be observed at Myawaddy in the southeast of Kayin State on the Thai–Myanmar border (~5%), as well as Loikaw and Demoso in the northern part of Kayah (~5 to 6%). Similar spatial patterns of malaria risk were observed in separate joint models of *P. falciparum* and *P. vivax* transmission, with the overall prevalence of *P. vivax* being higher than *P. falciparum* (*SI Appendix*, Discussion 4).

## Discussion

Our study advances current approaches to surveillance of malaria transmission by developing a Bayesian geostatistical modeling framework that allows joint modeling of serological and molecular biomarkers measured in samples collected during routine surveillance by village health volunteers. Our joint model affirms the (positive) relationship between SG6 and malaria transmission intensity (as captured by CSP positivity and PCR prevalence), providing evidence that these vector and parasite serological biomarkers may serve as suitable alternative metrics with which to perform surveillance for malaria and its vectors. This framework combines a sensitive detection methodology, applied in routine surveillance systems, and fine-scale predictive modeling to address a critical need to improve the tools we use to measure malaria transmission and perform surveillance. Having granular (1 km × 1 km) predictions of malaria risk is highly important in settings approaching elimination, where the stratification of malaria case data determined at the regional or township level to guide policy (25) is insufficient to accurately predict malaria risk given the heterogeneity in malaria infections and reliance on insensitive conventional diagnostic methods [such as rapid diagnostic tests (RDTs) and microscopy].

Using an ecological modeling framework, our study quantifies the associations with environmental and climatic variables that are risk factors for *Anopheles* occurrence and *Plasmodium* spp. infections and predicts probabilistic maps of anti-SG6 IgG seroprevalence. We show that the seroprevalence of antibodies against *Anopheles* salivary SG6 was moderate-to-high [similar to the other serological investigation from Myanmar (26)] and our maps quantify fine-scale (1 km × 1 km) spatial and temporal heterogeneity in exposure to *Anopheles* bites across the region. Our predictive maps improve upon the commonly referenced Malaria Atlas Project entomological maps which, given the dearth in entomological surveys that collect vector endpoints (i.e., occurrence, HBR, EIR), predict only the occurrence of the global dominant vector species on a static macroscale (27–31) and do not capture either the abundance of the vector nor human exposure to vector bites per se. Yet diversity in the host-seeking, feeding, and resting behaviors of the mosquitoes (27, 32, 33), across small spatial scales (34, 35), as well as in human behaviors [e.g., proximity to forested areas/water, occupational exposure, land use, intervention coverage, intervention usage, migration, etc. (36–38)], and the microclimatic environment they exist in (e.g., temperature, precipitation, vegetation, topography, etc.) all contribute to the complex vector-human interaction (39), and are therefore imperative to the measurement of exposure to vector bites. The fine-scale heterogeneity in SG6 observed in our study may reflect significant variation in exposure to *Anopheles* biting rates across small spatial scales as reported in several entomological surveys from sites across the Asia Pacific (9, 26, 35, 40–42). Having granular predictions of the interaction between human and vector populations through use of a serological biomarker of *Anopheles* biting exposure as shown here, could be useful in intervention planning exercises to identify hot spots of ongoing transmission, as well as populations receptive to malaria transmission, or areas with gaps in coverage or ineffective usage of core interventions (i.e., long-lasting insecticide-treated bed nets and indoor residual spraying) that require supplementary interventions to reduce exposure to *Anopheles* bites (e.g., personal repellent, insecticide-treated hammocks, spatial repellents, outdoor residual spraying).

Using a stepwise model selection procedure of environmental and climatic variables associated with vector occurrence, we found that increasing anti-SG6 seroprevalence was associated with increasing rainfall, potential evapotranspiration, and similarity between day-night temperatures. Our study also demonstrated that anti-SG6 IgG antibodies were dynamic over time, with the lowest overall seroprevalence of anti-SG6 antibodies in the rainy season when vector abundance would be assumed to be the highest (Fig. 1 and *SI Appendix*, Fig. S3). This may seem counterintuitive; however, we observe that anti-SG6 antibodies begin to decline at the end of the hot and start of the rainy season, before steadily increasing in the last 3 mo of the rainy season and are then sustained at high levels throughout the remainder of the study. Peaks of anti-*Anopheles* salivary antibodies at the end of the rainy season have also been observed in cross-sectional surveys in Africa (43). This delayed seasonal effect is most likely due to the biology of the dominant vectors of the region: while we are limited in our ability to compare this directly as entomological endpoints were not measured in our study, a lag between the onset of the rainy season (May) and the peak densities of the dominant vectors has been previously reported in studies from Myanmar, with *An. dirus s.l.* and *An. minimus s.l.* peaking in September to October, and November to December, (end of rainy and beginning of cool seasons) respectively (33, 44) (*SI Appendix*, Fig. S2). While there are limited entomological data specific to the dynamics of these vectors in the Myanmar context, this may be due to a natural delay in the time it takes for these vectors to make use of newly formed waterbodies for oviposition, larval development, and emergence [larval to pupal development time found to be 13.5 to 15.6 d for *An. minimus* (45) and 8 to 13 d under laboratory conditions for *An. dirus* (46)] or could perhaps be a result of the heavy rains experienced in July 2015 flushing away larval habitats of *An. dirus s.l.* [reported in some studies from the GMS (47–49)]. The boosting and decay dynamics of these antibodies in response to *Anopheles* biting exposure will also contribute to seasonal patterns but are yet to be fully elucidated—most studies investigating seasonality are biannual or at most quarterly (50–52) and our study of 15 mo duration could not capture the dynamics of anti-*Anopheles* salivary antibodies in response to repeated seasonal changes in *Anopheles* biting exposure. We were unable to model the longitudinal dynamics of these antibodies in a spatiotemporal framework in the present study due to low monthly testing rates in large numbers of villages; however, future studies could further explore these longitudinal and potentially lagged dynamics with more temporally resolved data. However, our analyses using seasonally partitioned data showed that spatial patterns of anti-SG6 IgG antibodies were similar regardless of seasonality (despite seasonal differences in the overall prevalence). This suggests that surveys, or surveillance through routine active and passive case detection, could be performed year-round to identify areas with the greatest *Anopheles* biting exposure that could be targeted with appropriate vector control interventions.

While two other studies have measured an association between antibodies against both sporozoite and *Anopheles* salivary proteins [one showing a weak correlation (53) and the other showing a strong dose-dependent relationship (52)], however they did not combine these metrics or compare them to malaria outcomes. By including them in a joint Bayesian geostatistical model, we have leveraged CSP positivity to learn the relationship between SG6 and PCR prevalence and present this as a framework to perform serosurveillance of malarial transmission intensity. This framework provides a sensitive and logistically feasible proxy measure for EIR, answering the World Health Organization’s call for innovative tools and improved approaches to enhance entomological surveillance capacity (54). While we are unable to compare directly against entomological endpoints due to scarce and geographically sparse entomological data, a comparison of our map of the predicted distribution of PCR-detectable *Plasmodium* spp. infections and the Malaria Atlas Project maps of the parasite rates for *P. falciparum* [in 2 to 10 y olds (*Pf*PR_2-10_)] and *P. vivax* [in 1 to 99 y olds (*Pv*PR_1-99_)] from the same time period (mostly detected using RDT or microscopy and similarly used to define malarial endemicity) (10) show somewhat different spatial patterns and “hot spots” of malaria transmission (i.e., areas with greater than average malaria prevalence). Overall, our map indicates a higher prevalence of malaria infections across Bago (East), Kayah and Kayin (mean: 3.6%) compared to estimates of *Pf*PR_2-10_ (mean: 0.6%) and *Pv*PR_1-99_ (mean: 1.1%). This highlights a key advantage of a serological and molecular approach to surveillance, by effectively targeting a higher prevalence point (i.e., identifying recently cleared or low-density infections, respectively) it may help overcome sensitivity limitations of both traditional entomological and malarial case surveillance to detect the few positive cases in settings approaching elimination (55). This is also evidenced by a study in Kayin state that identified that 66% of *P. falciparum* and 96% of *P. vivax* infections detected using ultrasensitive PCR were missed by RDTs (56). Similar to our map of malaria prevalence, the Malaria Atlas Project maps indicate hot spots of *P. falciparum* and *P. vivax* infections in the central and southern regions of Bago (East) respectively. However, the Malaria Atlas Project maps (10) and the stratification of this ultrasensitive PCR incidence data for village tracts in Kayin (56) also indicate a large hot spot of *P. vivax* infections in east Kayin along the Thai-Myanmar border that is not evident on our map of *Plasmodium* prevalence (although their prevalence estimates do not exceed ours of *Plasmodium* spp. prevalence and our model uncertainty for this area is large due to a lack of observed data in this region). One possible explanation for this is a tendency for parasite prevalence surveys to overestimate ongoing *P. vivax* transmission, as blood-stage infections can be caused by relapses from dormant liver stages rather than through the bites of infective mosquitoes. This is reflected in our data where the longitudinal trends of anti-CSP approximately followed *P. falciparum* but not *P. vivax* prevalence. Combining parasite detection with a serological marker for vector exposure may therefore allow us to improve surveillance of the transmission of *P. vivax* malaria by identifying newly transmitted infections.

The present study measures antibodies specific to vector and parasite antigens in samples collected during routine surveillance by an established network of village health volunteers providing malaria services in hard-to-reach villages in Southeast Myanmar. As the cornerstone of the current surveillance strategy in most malaria-endemic countries, this expansion of routine malaria testing provided by village health volunteers into a sensitive and feasible surveillance platform to quantify *Anopheles* biting exposure and malaria transmission represents a key strength of this study. Such a serosurveillance platform could employ a similar sample collection framework to this study, or perhaps involve elution of serum from used RDTs or serological point-of-contact tests (i.e., SG6 and CSP), which would require limited additional capacity and training for expansion into current community-based malaria programs. Indeed, incorporation of data collected by public health surveillance systems into species distribution models for vector surveillance and mosquito-borne disease control has been identified as an underutilized avenue for estimating spatial risk (57). One possible limitation of applying this approach is that measurements will be collected and geolocated in the villages where participants reside and may not accurately reflect where participants are exposed to *Anopheles* bites (i.e., in the forest), potentially explaining why we identified high-risk areas along transport routes. This may also contribute to the counterintuitive negative association we observed between anti-*Anopheles* salivary antibodies and tree coverage and proximity to water (e.g., villages are of-themselves less likely to be densely forested). However, in settings such as the GMS where malaria services and interventions are ultimately delivered by village health volunteers, quantification of the molecular and serological profile of all high-risk populations that reside in villages (regardless of transmission within the village) is beneficial. To address this potential limitation, future work could explore defining a buffer zone around each village as a way to capture nearby forested areas and water sources that may be sites of exposure to vector bites. Our findings of similar spatial patterns and hot spots of anti-SG6 IgG seropositivity in our preliminary investigations using samples collected from village residents and high-risk participants (migrants and forest-goers) imply that we could improve the efficiency of this surveillance by targeting sample collection in either group.

The validity of our geospatial analysis relies on the accurate measurement of exposure to *Anopheles* bites and the accuracy of GPS coordinates. We used antibodies against SG6 derived from the dominant African vector *An. gambiae* (gSG6), the most commonly investigated *Anopheles* biomarker. While this species is not present in the GMS, it shares 52 to 78% sequence identity to SG6 in GMS *Anopheles* spp. vectors (58), and previous studies in the GMS have shown *An. gambiae* SG6 antibodies to be correlated with HBR (total *Anopheles* population and primary malaria vectors) (26). However, a species-specific salivary antigen approach may allow us to further improve granularity in our estimates of *Anopheles* biting exposure, highlighting an important area for further research. The approximation of location by matching village names to existing place codes (Pcodes) from the Myanmar Information Management Unit and their associated GPS coordinates is a potential limitation of our study, as it may cause some error in the estimated associations between anti-SG6 IgG seroprevalence and our environmental covariates, and subsequently impact the predicted probability of our outcomes of interest. However, the validation procedures for our generated model estimates indicate a good model fit with good agreement between the observed and predicted values of anti-SG6 IgG seroprevalence. As our study is an opportunistic spatial analysis of samples collected as part of a larger trial, our study has two potential limitations. First, as entomological endpoints are not routinely collected and were not measured in this study, we are limited in our ability to directly compare our predictions against metrics of vector exposure (HBR) and malaria transmission (EIR). Second, the clustered nature of these villages’ results in high levels of uncertainty in our predictions and a tendency toward the mean value across large areas of the region of interest (particularly where observed data are scarce). To address these limitations, additional studies employing the same Bayesian geospatial modeling framework reported here that directly measure entomological endpoints (both HBR and EIR) should further investigate the external validity of study findings including their generalizability across a range of transmission settings. Beyond the usual principles of spatial design for prediction that favor a regular/uniform coverage over the area of interest augmented with multiscale focal sampling to assist with hyperparameter estimation (59), one would want to consider heavier sampling around the start of the transmission season before the anti-SG6 prevalence becomes “saturated.” Given that adaptive sampling designs are increasingly being studied in disease mapping (60, 61), it is worth noting that, if logistically feasible, this could even be adaptive with the start of the heavier sampling period triggered by exceedance of a given seroprevalence threshold [e.g., Charney, Kubel, and Eiseman (62)].

The geospatial joint modeling framework of vector and parasite serological biomarkers we developed to predict malaria transmission intensity without necessitating laborious entomological investigations is a key strength of our study. An accurate characterization of the relationship between climate and mosquito biting rates, and its modulation by human behaviors and other factors, are essential parameters for forecasting the impacts of climate change on vector-borne disease. The hierarchical structure of our model makes clear the separation of effects into each of these components, namely the directly represented contributions of environment and of each metric on the others and the indirectly represent contributions of unobserved influences at different stages of the transmission system via the spatial random effects. While we have focused here on prediction and have not attempted to propose a causal structure for the influence of the environmental covariates, the directed graph structure introduced here for the relationships between the available biomarkers facilitates a causal interpretation at this level against which the plausibility of the learned relationships can be judged. The cross-validation results of this modeling approach and the recovery of positive associations between the biomarkers examined despite the limitations of sample size and spatial coverage in this study confirm this as a promising approach for future studies supporting risk mapping with larger serological surveys.

## Conclusions

Our study presents predictive maps of the seroprevalence of antibodies against *Anopheles* salivary antigen SG6, identifying spatial and temporal microheterogeneity in exposure to vector bites, advancing on previous static macromaps of only vector occurrence. Furthermore, we identified foci of ongoing malaria transmission by developing a joint modeling approach that combines vector and transmission parasite serology measured in samples collected by village health volunteers, highlighting a potential framework to enhance entomological and malaria transmission surveillance capacity that could be readily incorporated into existing routine surveillance networks. We show that these antibodies can serve as sensitive, accurate, and feasible tools for the surveillance of malaria transmission, potentially helping to overcome sensitivity limitations associated with detecting the few malarial-infected vectors and individuals in settings approaching elimination. More granular stratification of malaria risk could be used for targeting appropriate vector control and malaria elimination interventions to areas with the greatest need, which will ultimately help accelerate progress toward elimination.

## Materials and Methods

This study uses data from a stepped-wedge cluster randomized controlled trial assessing the effectiveness of personalized insect repellent delivered by village health volunteers performing routine malaria services in 114 villages in Southeast Myanmar during 2015 to 2016 (63). While the overall effect of repellent was found to be protective against *P. falciparum* infection, there was significant heterogeneity in the prevalence *Plasmodium* spp. infections at the village level (64). Informed consent was collected from all participants or parents/guardians, and the study protocol was approved by ethics committees from the Government of the Republic of the Union of Myanmar Ministry of Health Department of Medical Research (21/Ethics/2015) and the Alfred Hospital Ethics Committee (95/15).

### Study Area.

The study was performed in 114 hard-to-reach villages from three states [Kayin, Kayah, and Bago (East)] in Southeast Myanmar. Geolocations (longitude and latitude) of villages were determined retrospectively (procedure outlined in *SI Appendix*, *Methods*) and were available for 104 villages (*SI Appendix*, Fig. S17).

### Vector, Malaria, and Serology Data.

Participants receiving routine malaria testing from village health volunteers were invited to provide a dried blood spot (DBS) for molecular detection of parasitemia and immunoassays (13,594 DBS samples were collected from 29,132 routine tests). DNA was extracted, and *P. vivax* and *P. falciparum* were detected using a duplex qPCR as previously reported (64) (916 samples not available due to insufficient sample for DNA extraction). IgG to recombinant *P. falciparum* (3D7) and *P. vivax* (210) CSP (*Pf*CSP and *Pv*CSP, expressed in HEK 293 cells) (65) was measured by a high-throughput enzyme-linked immunosorbent assay (ELISA) performed on a liquid handling robot (JANUS automated work station, Perkin Elmer), using an established protocol previously described (66) (1,231 samples not available for *Pv*CSP assay due to sample exhaustion). IgG to the synthetic *An. gambiae* gSG6-P1 peptide (Genscript, USA) was measured by the high-throughput ELISA protocol, with some modifications (outlined in *SI Appendix*, *Methods*). Seropositivity thresholds were determined for each antigen, defined as standardized optical density (OD) above the mean +3SD of the negative controls (from Melbourne, Australia).

### Environmental and Spatial Data.

We identified several satellite-derived spatial, environmental, and climatic covariates that we hypothesized to be related to vector exposure and malaria transmission (described in Table 1). All covariate rasters were resampled to 1 km × 1 km resolution and were standardized and centered to have a mean of zero and a SD of one. The variance inflation factor (VIF) function from the *car* R package was used to identify multicollinearity between variables, excluding variables with VIF estimates >10. Models were first fitted separately to assess univariate associations between our vectorial, climatic, and environmental covariates and our outcome before undergoing stepwise covariate selection using Watanabe–Akaike Information Criterion and Deviance Information Criterion (67, 68) to identify the most parsimonious model.

### Statistical Analysis.

A Bayesian hierarchical geostatistical model was fitted to data of the seroprevalence of antibodies against *Anopheles* salivary protein SG6, using the integrated nested Laplace approximation for model inference and prediction.

Let $Y_{l}$, $n_{l}$, and $p_{l}$ be the number of infected individuals, the number of individuals screened, and the seroprevalence of anti-SG6 IgG antibodies at geocoded location *l* (*l* = 1, …, *N*). $Y_{l}$ is assumed to follow a binomial distribution:

$$Y_{l}∼Binomial\;(p_{l},n_{l}).$$

The seroprevalence of anti-SG6 IgG antibodies, $p_{l}$, is represented with a structured additive regression model with a generalized linear predictor on the logit scale:

$$logit\left(p_{l}\right)=\alpha +X_{l}\beta +\zeta_{l},$$

where α is the intercept, $X_{l}$ is a matrix of covariates, β is the corresponding regression coefficients, and $\zeta_{l}$ are spatial random effects modeled using a zero-mean Gaussian Markov random field with a Matérn covariance function.

We also present a Bayesian hierarchical geostatistical joint model with multiple likelihoods fitted to the seroprevalence of biomarkers of vector (SG6) and malaria (CSP) exposure and malaria prevalence (PCR) outlined below.

Let $Y_{l}^{SG6}$, $Y_{l}^{\mathrm{CSP}},$ and $Y_{l}^{\mathrm{PCR}}$ denote the numbers of SG6 and CSP seropositive and PCR *Plasmodium* spp. positive individuals respectively, observed among $n_{l}$ individuals tested at location *l* (*l* = 1, …,*N*). Each is assumed to have a binomial sampling distribution conditionally independent given the latent seroprevalences and parasite prevalence (namely $p_{l}^{SG6}$, $p_{l}^{\mathrm{CSP}}$ and $p_{l}^{\mathrm{PCR}}$) at each location, i.e.,

$$Y_{l}^{SG6}∼Binomial\;(p_{l}^{SG6},n_{l}),$$

$$Y_{l}^{\mathrm{CSP}}∼Binomial\;(p_{l}^{\mathrm{CSP}},n_{l}),$$

$$Y_{l}^{\mathrm{PCR}}∼Binomial\;(p_{l}^{\mathrm{PCR}},n_{l}),$$

These latent prevalences are modeled jointly in a multivariable spatial field form with the following hierarchical structure:

$$logit\left(p_{l}^{SG6}\right)=\alpha^{SG6}+X_{l}\beta^{SG6}+\zeta_{l}^{SG6},$$

$$logit\left(p_{l}^{\mathrm{CSP}}\right)=\alpha^{\mathrm{CSP}}+X_{l}\beta_{SG6}^{\mathrm{CSP}}(logit\left(p_{l}^{SG6}\right))+\zeta_{l}^{\mathrm{CSP}},$$

$$logit\left(p_{l}^{\mathrm{PCR}}\right)=\alpha^{\mathrm{PCR}}+X_{l}\beta_{SG6}^{\mathrm{PCR}}\left(logit\left(p_{l}^{SG6}\right)\right)+X_{l}\beta_{\mathrm{CSP}}^{\mathrm{PCR}}\left(logit\left(p_{l}^{\mathrm{CSP}}\right)\right)+\zeta_{l}^{\mathrm{PCR}},$$

with $\zeta_{l}^{SG6}$, $\zeta_{l}^{\mathrm{CSP}},$ and $\zeta_{l}^{\mathrm{PCR}}$ each representing independent spatial random effects modeled with zero-mean Gaussian Markov random field with a Matérn covariance function; the hyperparameters of each are constrained with independent priors (and jointly optimized by log posterior density for an empirical Bayes approximation).

**Fig. 4.**
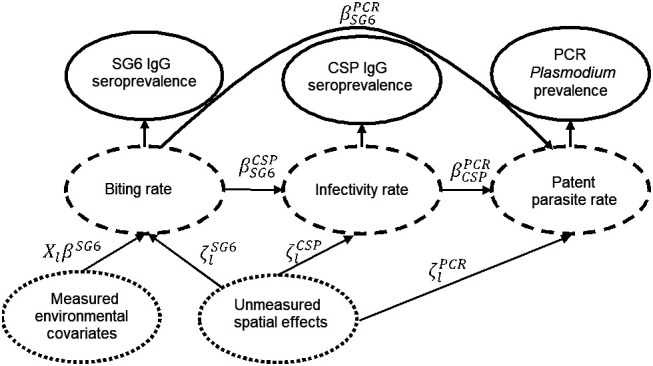
Directed acyclic graph of the joint model. Dotted circles represent covariates, dashed circles represent latent outcome variables, and solid circles represent measured outcome variables.

Intuitively, this model supposes the biting rate is the primary cause of spatial variation in all these exposure markers, with the environmental covariates directly influencing SG6 prevalence, SG6 prevalence then influencing CSP prevalence and both influencing PCR prevalence. Within the logit space transformation, linear relationships are assumed for these influence functions for simplicity of interpretation, while spatially correlated offsets are added to limit exposure to misspecification of these relationships. The model structure was chosen to favor Bayesian shrinkage toward a simple correlation of these three metrics, although it is worth noting that the directed acyclic graph (DAG; Fig. 4) representation of this model suggests also a causal interpretation consistent with the intuitive framing above.

Validation analyses to assess the models’ goodness of fit and predictive accuracy were performed by Pearson correlation of observed versus predicted data and hold out procedures, respectively. Specifically, the models were trained using observed data from a subset of 90% of the villages and then used to predict the prevalence in the withheld villages (i.e., the test dataset, 10% of villages). This process was repeated 20 times with different subsets, and the observed vs. predicted prevalence was compared (using Pearson correlation) for each omitted site in the test dataset and for a series of bins (deciles) by the predicted prevalence.

## Supplementary Material

Appendix 01 (PDF)

## Data Availability

Data cannot be made publicly available because it would breach compliance with the ethical framework of the Ethics Review Committee on Medical Research Involving Human Subjects, Department of Medical Research, Myanmar Ministry of Health and Sports. Deidentified individual participant data are stored on Burnet Institute servers and will be made available from the corresponding author (freya.fowkes@burnet.edu.au) to applicants who provide a sound proposal to The Ethics Review Committee on Medical Research Involving Human Subjects, Department of Medical Research, Myanmar Ministry of Health and Sports (No. 5 Ziwaka Road, Dagon PO Yangon, Myanmar; (+95) 01 375447 extension 118; ercdmr2015@gmail.com) contingent of their approval.
