# Supplementary material for: Geospatial joint modeling of vector and parasite serology to microstratify malaria transmission
Source: Proc Natl Acad Sci U S A. 2024 Jun 4;121(24):e2320898121. doi: 10.1073/pnas.2320898121 (PMC11181033; doi:10.1073/pnas.2320898121)
Supplement: Supplementary file 1 — Appendix 01 (PDF) [file pnas.2320898121.sapp.pdf]

Supporting Information for

Geospatial joint modeling of vector and parasite serology to micro-stratify malaria transmission

Ellen A Kearney, Punam Amratia, Su Yun Kang, Paul A Agius, Kefyalew Addis Alene, Katherine O'Flaherty, Win Han Oo, Julia C Cutts, Win Htike, Daniela Da Silva Goncalves, Zahra Razook, Alyssa E Barry, Damien Drew, Aung Thi, Kyaw Zayar Aung, Htin Kyaw Thu, Myat Mon Thein, Nyi Nyi Zaw, Wai Yan Min Htay, Aung Paing Soe, James G Beeson, Julie A Simpson, Peter Gething, Ewan Cameron, Freya JI Fowkes*

Freya JI Fowkes
Email: freya.fowkes@burnet.edu.au

This PDF file includes:

Supporting text

Figures S1 to S17

Tables S1 to S6

Table S1. Participant demographics and Plasmodium spp. infection and serology status, by state.

	Bago (East)	Kayah	Kayin	Total
**No. of samples**	N=1,147	N=3,872	N=8,575	N=13,594
**No. of villages, (n)**	10	38	56	104
**Sex, % (n)**				
Male	44.0% (505)	50.5% (1,954)	51.5% (4,412)	50.5% (6,871)
**Risk group, % (n)**				
Resident	46.2% (530)	31.6% (1,225)	47.1% (4,042)	42.6% (5,797)
Migrant	11.2% (129)	2.8% (108)	13.9% (1,196)	10.5% (1,433)
Resident/Forest Dweller	42.5% (488)	65.6% (2,539)	38.9% (3,337)	46.8% (6,364)
**Age, Median (IQR)**	25.0 (17.0-40.0)	20.0 (10.0-35.0)	18.0 (9.0-32.0)	19.0 (10.0-35.0)
**PCR, % (n)**	N=1052	N=3552	N=8074	N=12678
P. vivax	1.3% (15)	1.6% (61)	1.4% (122)	1.5% (198)
P. falciparum	1.2% (14)	0.8% (30)	0.9% (76)	0.9% (120)
Mixed	0.7% (8)	0.6% (24)	0.6% (54)	0.6% (86)
**Anti-SG6 IgG**				
OD, Median (IQR)	2.1 (1.5-2.4)	2.1 (1.5-2.4)	2.0 (1.4-2.4)	2.1 (1.4-2.4)
Seroprevalence, % (n)	62.3% (715)	63.6% (2,464)	57.1% (4,898)	59.4% (8,077)
**Anti-*Pf*CSP IgG**				
OD, Median (IQR)	0.3 (0.1-0.7)	0.3 (0.1-0.7)	0.3 (0.1-0.7)	0.3 (0.1-0.7)
Seroprevalence, % (n)	18.8% (216)	19.0% (737)	18.3% (1,567)	18.5% (2,520)
**Anti-*Pv*CSP IgG**	N=1037	N=3538	N=7788	N=12363
OD, Median (IQR)	0.6 (0.3-0.9)	0.5 (0.3-0.9)	0.6 (0.3-0.9)	0.6 (0.3-0.9)
Seroprevalence, % (n)	19.0% (197)	16.2% (574)	19.6% (1,527)	18.6% (2,298)

Abbreviations: IQR – interquartile range (indicated by the $25^{th}$and 75th percentiles), PCR – polymerase chain reaction, OD – optical density.

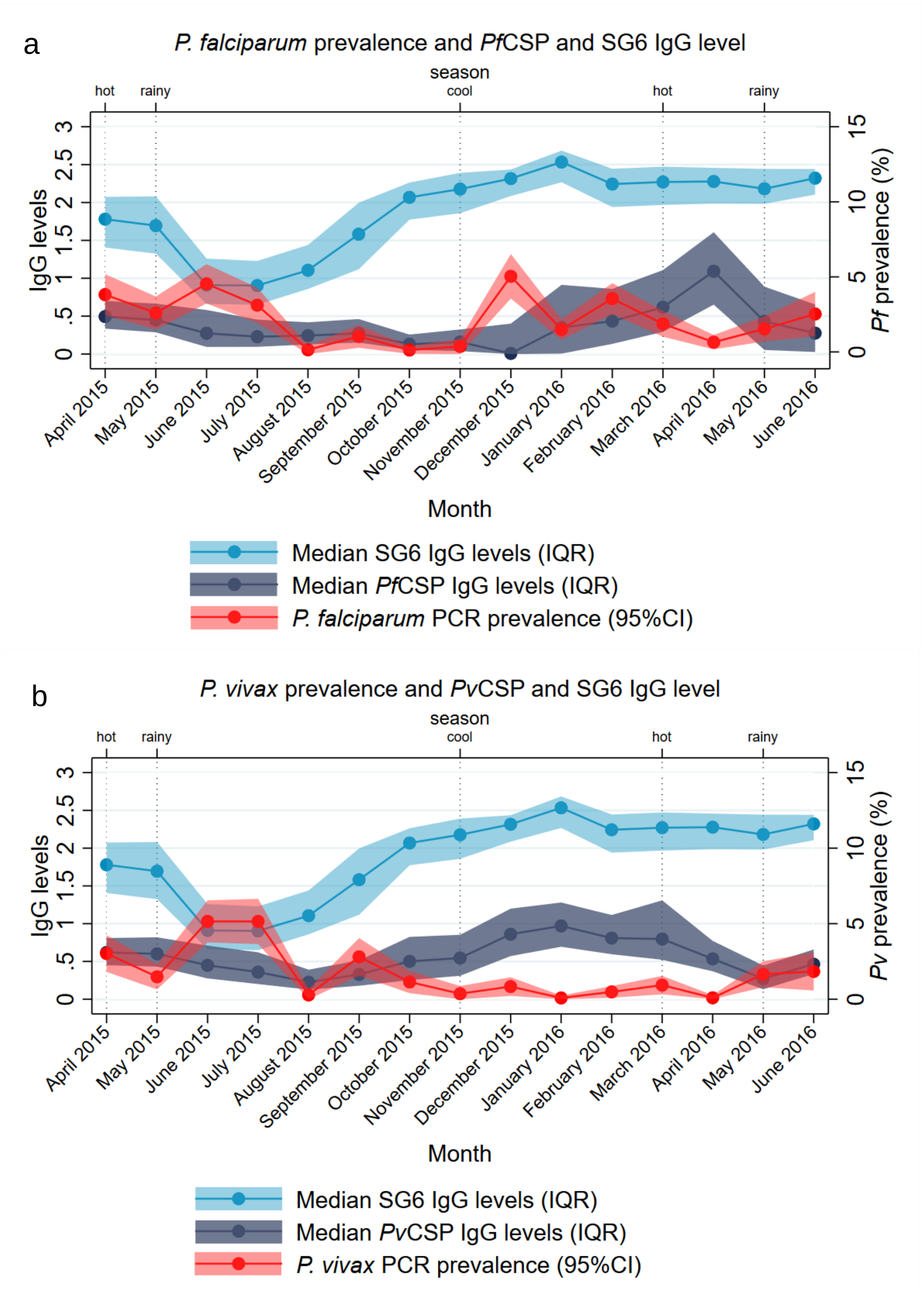

Fig. S1. Overall anti-SG6 IgG levels, (a) *P. falciparum* and (b) *P. vivax* transmission dynamics (prevalence and anti-CSP IgG) over time. (a) shows the median (IQR) levels of IgG to *P. falciparum* transmission-stage (*Pf*CSP) and the vector salivary (SG6) antigens (left y-axis), as well as the prevalence (95%CI) of *P. falciparum* infection (right y-axis), over the 15-month study period. (b) shows the median (IQR) levels of IgG to *P. vivax* transmission-stage (*Pv*CSP) and the vector salivary (SG6) antigens (left y-axis), as well as the prevalence (95%CI) of *P. vivax* infection (right y-axis), over the 15-month study period. Vertical dotted lines indicate season.

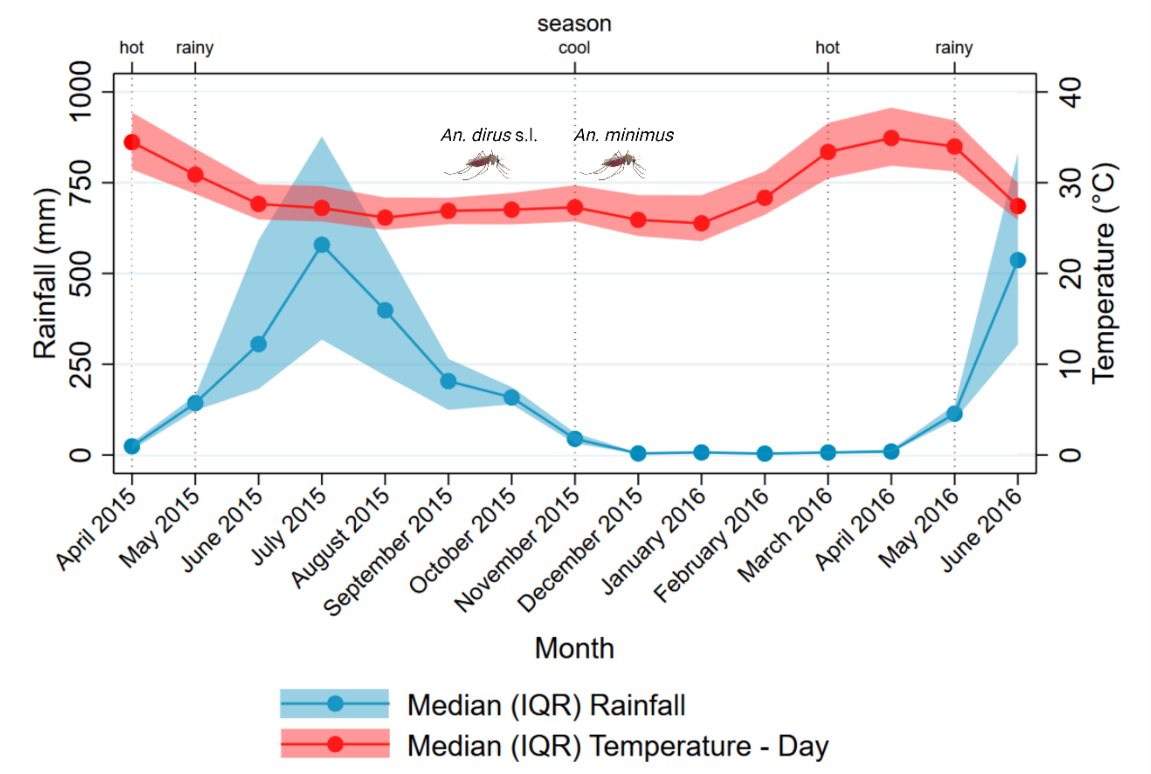

Fig. S2. Monthly rainfall (mm) and temperature (ºC) for study area. Figure shows the median and interquartile range (IQR) of rainfall (56) (left y-axis) and day-time land surface temperature (57) (right y-axis) for the study region over time. Vertical dotted lines indicate typical seasonal patterns with mosquito icons (from BioRender) indicating the peak densities of *Anopheles dirus sensu lato* (s. l.) and *An. minimus* (the dominant vector species of Myanmar) as reported in Oo, Storch and Becker (29) and Suwonkerd*, et al.* (18).

Supporting Information Discussion 1

Seasonal anti-SG6 IgG seroprevalence for Bago (East), Kayah and Kayin.

As we had access to samples collected over time, we sought to define heterogeneity in anti-SG6 IgG seroprevalence both spatially and temporally. We were prevented from fitting a spatiotemporal that included an autoregressive correlation structure to properly account for temporal dynamics due to low rates of monthly sampling in each village. Instead, data were partitioned by season and a geostatistical model using the covariates identified as important in the non-temporal model (regression coefficients shown in Table S2) was fitted to each dataset separately. Fig. S3 shows the predicted posterior mean seroprevalence of anti-SG6 IgG for the hot, rainy and cool seasons, with the standard deviation of the pixel-wise predicted probability as an indication of uncertainty in our model shown in Fig. S4. Interestingly, the seroprevalence of anti-SG6 IgG antibodies is markedly different depending on seasonality (Fig. S3), with the median seroprevalence dropping significantly from the hot (65%; range: 10-97%) to the rainy (35%; range: 19-60%) season, before increasing to higher levels during the cool season (81%, range: 41-97%). However, the patterns of spatial heterogeneity in anti-SG6 IgG seroprevalence are similar between each season and when considered altogether.

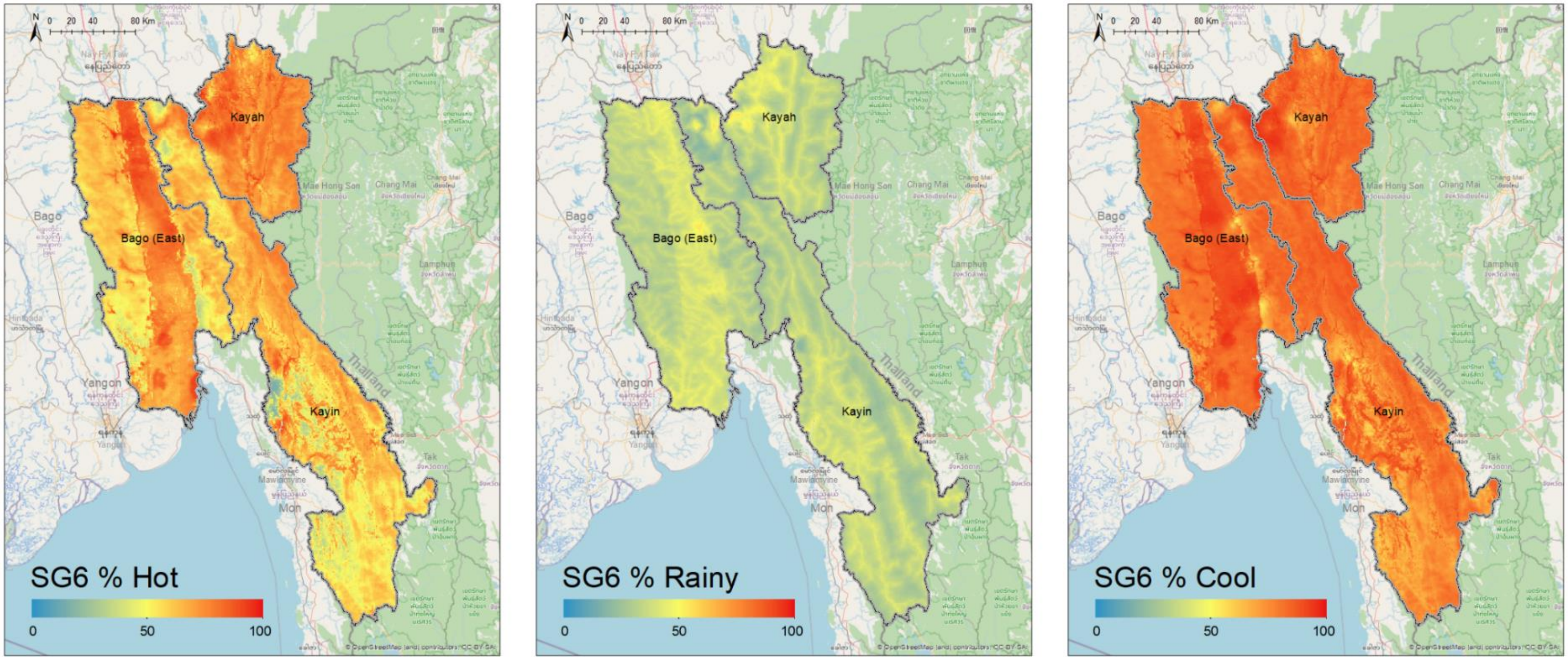

Fig. S3. Predicted anti-SG6 IgG seroprevalence for Bago (East), Kayah and Kayin, partitioned by season. Geospatial maps showing the predicted posterior mean seroprevalence of anti-SG6 IgG. Data were partitioned and models were fitted separately (hot: n=2,801; rainy: n=6,840; cool: n=3,953), with each model adjusting for rainfall, diurnal temperature difference, potential evapotranspiration, distance to water and tree coverage.

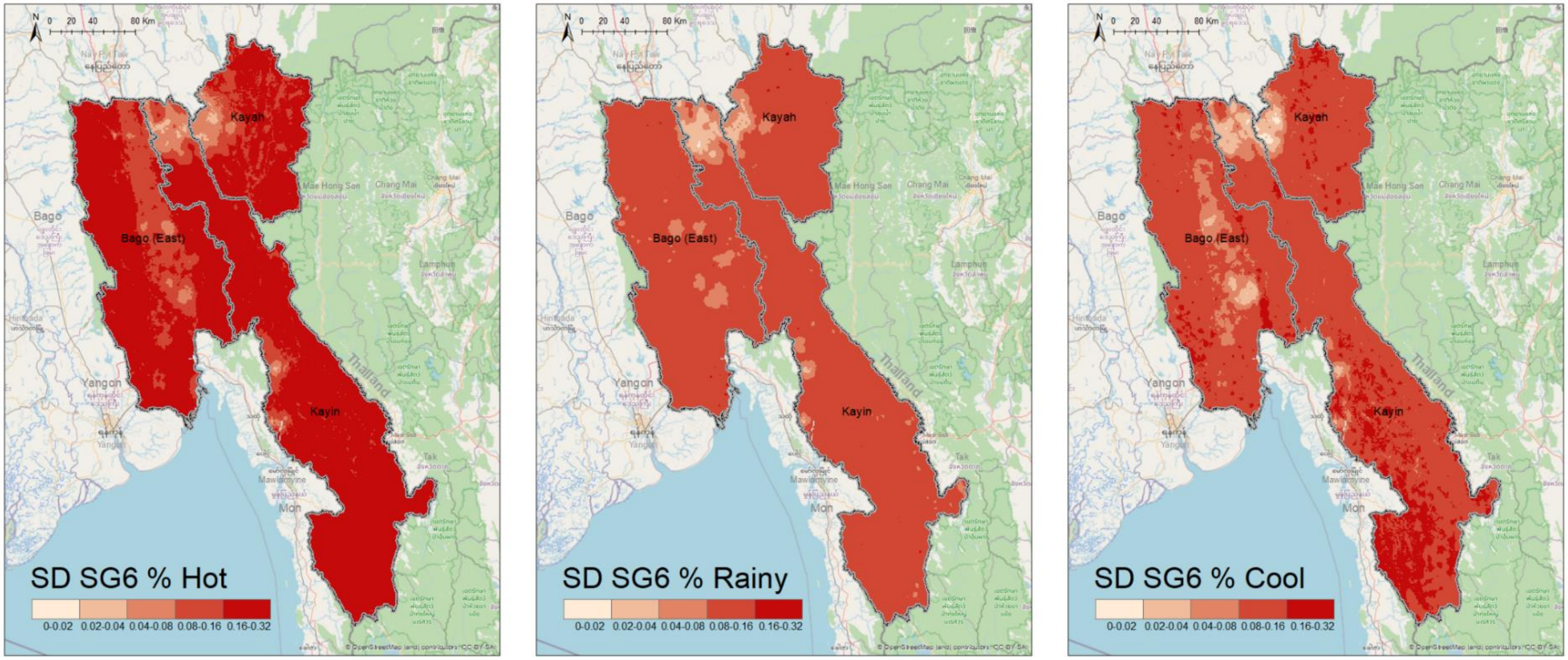

Fig. S4. Model uncertainty for predicted anti-SG6 IgG seroprevalence for Bago (East), Kayah and Kayin, partitioned by season. Geospatial maps showing the standard deviation in the predicted posterior mean probability of anti-SG6 IgG seropositivity. Data were partitioned and models were fitted separately, with each model adjusting for rainfall, distance to water, potential evapotranspiration, tree coverage and diurnal temperature difference.

Table S2. Regression coefficients from seasonally partitioned models of anti-SG6 IgG seroprevalence.

	Hot			Rainy			Cool		
	**OR**	**95% CrI**		**OR**	**95% CrI**		**OR**	**95% CrI**	
b0	2.26	1.35	3.81	0.78	0.60	1.02	5.99	4.19	8.57
Rainfall	0.74	0.48	1.15	1.12	0.88	1.43	0.77	0.56	1.06
Land Surface Temperature Diurnal Difference	0.57	0.38	0.87	1.02	0.78	1.32	0.68	0.45	1.04
Potential Evapotranspiration	1.11	0.76	1.61	0.96	0.77	1.18	0.91	0.64	1.30
Distance to water	1.44	0.94	2.21	1.01	0.78	1.30	1.12	0.72	1.73
Tree Coverage Fraction	0.46	0.28	0.75	0.81	0.61	1.10	0.48	0.31	0.74

Note. Table shows the odds ratios (OR) and 95% credible interval (95%CrI) from a Bayesian geostatistical model. Data from all villages were partitioned and models were fitted separately (hot: n=2,801; rainy: n=6,840; cool: n=3,953), with each model adjusting for covariates: rainfall, diurnal temperature difference, potential evapotranspiration, distance to water and tree coverage (scaled for a 1 standard deviation change).

Supporting Information Discussion 2

Anti-SG6 IgG seroprevalence for Bago (East), Kayah and Kayin by risk-group.

As our study uses samples collected as part of routine delivery of malaria services to residents and higher-risk individuals (migrants and forest-goers) living in hard-to-reach villages, we sought to determine any differences in the predicted distribution of anti-SG6 IgG seroprevalence by participant risk-group. While not usual to include individual-level demographic factors as covariates in geospatial models (largely due to a lack of this demographic data for out of sample areas), in these investigations we have partitioned our data by participant risk-group (low-risker: village residents vs higher-risk: migrants and forest-goers) and fit separate geostatistical models to each stratified dataset (with the same covariates identified in the overall model; coefficients shown in Table S3) in order to inform the appropriate populations to be targeted for surveillance in these hard-to-reach villages. Fig. S5 shows the predicted posterior mean seroprevalence of anti-SG6 IgG for our lower (a) and higher-risk (b) participants, with the standard deviation of the pixel-wise predicted probability as an indication of uncertainty in our model shown in Fig. S6.

The seroprevalence of anti-SG6 IgG antibodies was higher amongst high-risk populations (median: 54%; range: 28.8-87.1%) compared to our village residents (median: 27.6%; range: 4.6-75.7%) (Fig. S5). However, the patterns of spatial heterogeneity in anti-SG6 IgG seroprevalence are similar, with hot spots of higher SG6 seroprevalence in the northern parts of Bago (East), north-eastern Kayin and western Kayah evident in both village resident and higher risk participant analyses.

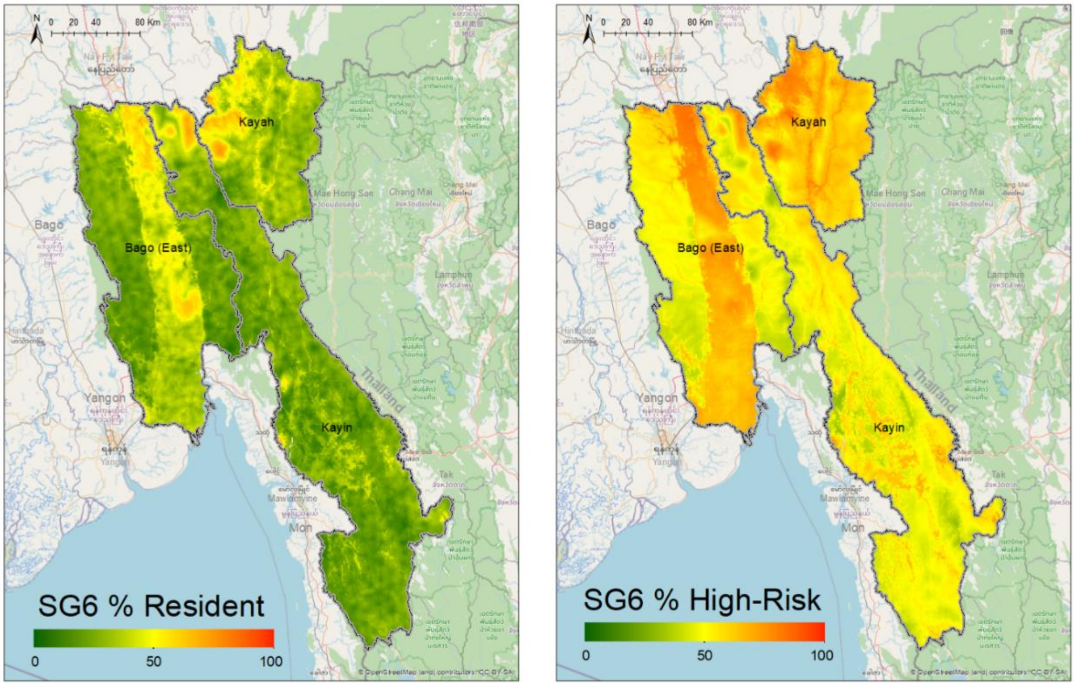

Fig. S5. Predicted anti-SG6 IgG seroprevalence for Bago (East), Kayah and Kayin, partitioned by participant risk group. Geospatial maps showing the predicted posterior mean seroprevalence of anti-SG6 IgG. Data were partitioned and models were fitted separately (lower-risk (village residents): n= 5,797; higher-risk (migrants and forest-goers): n=7,797), with each model adjusting for rainfall, diurnal temperature difference, potential evapotranspiration, distance to water and tree coverage.

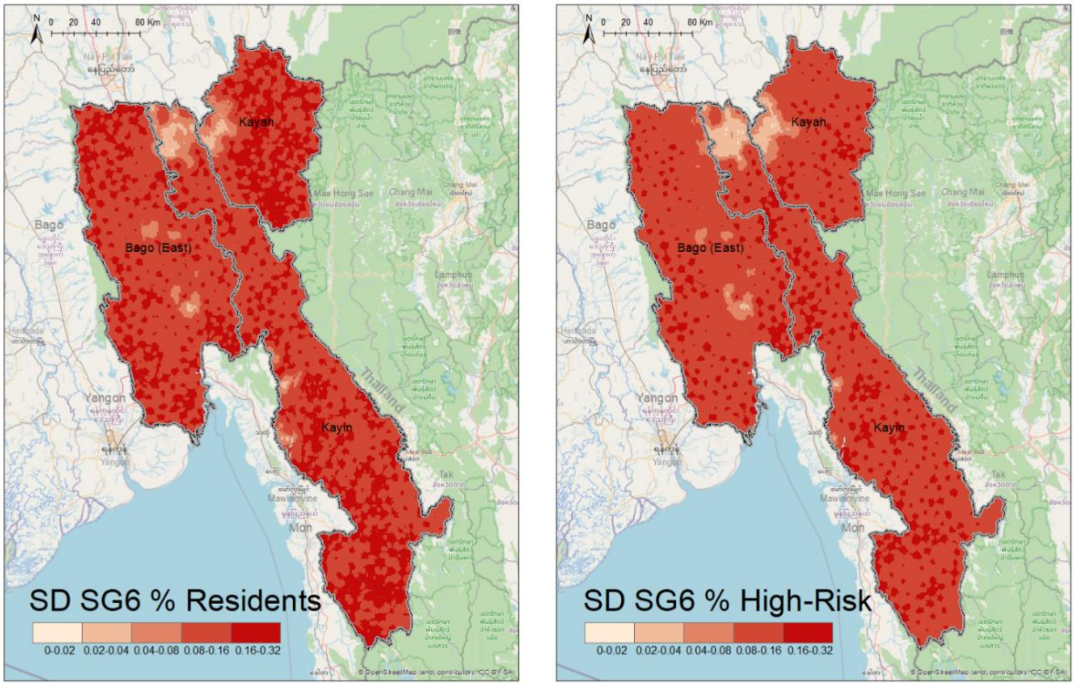

Fig. S6. Model uncertainty for predicted anti-SG6 IgG seroprevalence for Bago (East), Kayah and Kayin, partitioned by participant risk-group. Geospatial maps showing the standard deviation in the predicted posterior mean probability of anti-SG6 IgG seropositivity. Data were partitioned by risk-group (low-risk (village residents); high-risk (migrants and forest-goers)) and models were fitted separately, with each model adjusting for rainfall, distance to water, potential evapotranspiration, tree coverage and diurnal temperature difference.

Table S3. Regression coefficients and 95% credible intervals of covariates fitted in our geostatistical model of anti-SG6 IgG seropositivity, partitioned by participant risk-group.

	Low-risk			High-risk		
**Covariate**	**OR**	**95% CrI**		**OR**	**95% CrI**	
b0	1.46	1.07	2.00	1.35	1.00	1.81
Rainfall	0.81	0.62	1.07	0.77	0.59	1.00
Land Surface Temperature Diurnal Difference	0.73	0.57	0.95	0.97	0.77	1.21
Distance to Water	1.24	0.92	1.67	0.88	0.69	1.11
Potential Evapotranspiration	0.84	0.64	1.10	1.18	0.94	1.48
Tree Coverage Fraction	0.57	0.41	0.79	0.69	0.52	0.93

Note. Data given as odds ratio (OR) and 95% credible intervals (95%CrI) for a 1 standard deviation change in each covariate fitted in a Bayesian geostatistical model of the binomial response for the seroprevalence of anti-SG6 IgG antibodies, partitioned by risk-group (low-risk (village residents): n= 5,797; high-risk (migrants and forest-goers): n=7,797).

Table S4. Regression coefficients from joint model of SG6, CSP and PCR.

	OR	95%CrI	
Intercept SG6	1.28	0.71	2.31
Intercept CSP	0.36	0.32	0.41
Intercept PCR	0.04	0.04	0.05
Slope SG6	2.28	1.80	2.90
Slope CSP	1.13	0.81	1.56
Slope PCR	1.32	0.82	2.13
Distance to water	0.83	0.71	0.98
Topographical wetness index	1.06	0.94	1.20
Slope	0.89	0.83	0.96
Tree coverage fraction	0.61	0.46	0.80
Inaccessibility to cities	1.10	0.93	1.29
Night-time lights	1.20	1.03	1.40

Note. Table shows the odds ratios (OR) and 95% credible interval (95%CrI) from a Bayesian geostatistical joint model with multiple likelihoods. Models were fitted to participant data from all villages who had observations for each outcome (n=11,988), and adjusts for covariates: distance to water, topographical wetness index, slope, tree coverage fraction, inaccessibility to cities and night-time lights (scaled for a 1 standard deviation change).

Supporting Information Discussion 3

Model validation procedures for joint modelling of SG6, CSP and PCR-detectable Plasmodium spp. prevalence.

Here we describe the procedures employed to validate the novel joint modelling framework presented in Fig. 3. Fig. S7 shows a weak positive correlation between the observed SG6 and CSP IgG seroprevalence, and weak negative correlations between the observed prevalence of *Plasmodium* infections and anti-SG6 and CSP IgG seroprevalence. However, when using a joint model with multiple likelihoods, we observe shrinkage in the spread of the data and the emergence of a stronger association between anti-SG6 and CSP IgG, and weak positive correlations between *Plasmodium* prevalence and anti-SG6 and CSP IgG. Fig. S8 shows the models' goodness of fit, with the observed vs predicted mean probabilities of each of our outcomes at each village, and Fig. S9 and Fig. S10 show two kinds of model validation. The first, shown in Fig. S9, withholds the observed SG6, CSP and PCR data, and the second, shown in Fig. S10, withholds only the SG6 data. Allowing the model to fit to CSP and PCR data in the additional 10% of villages only marginally improved the model performance.

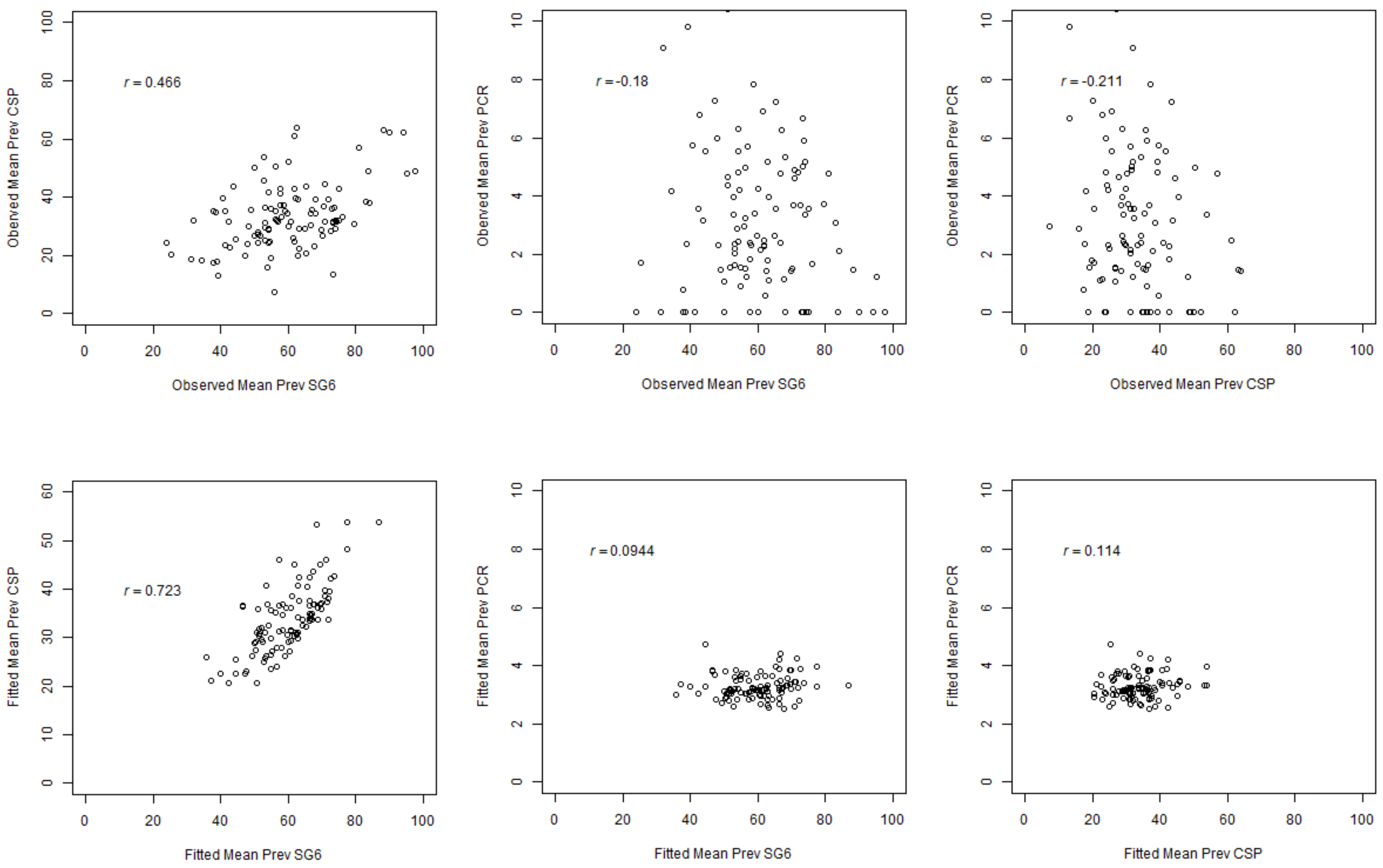

Fig. S7. Associations between outcomes of interest (SG6, CSP, PCR) in each village, both before (observed) and after (fitted) joint modelling. Pearson correlation used to estimate r.

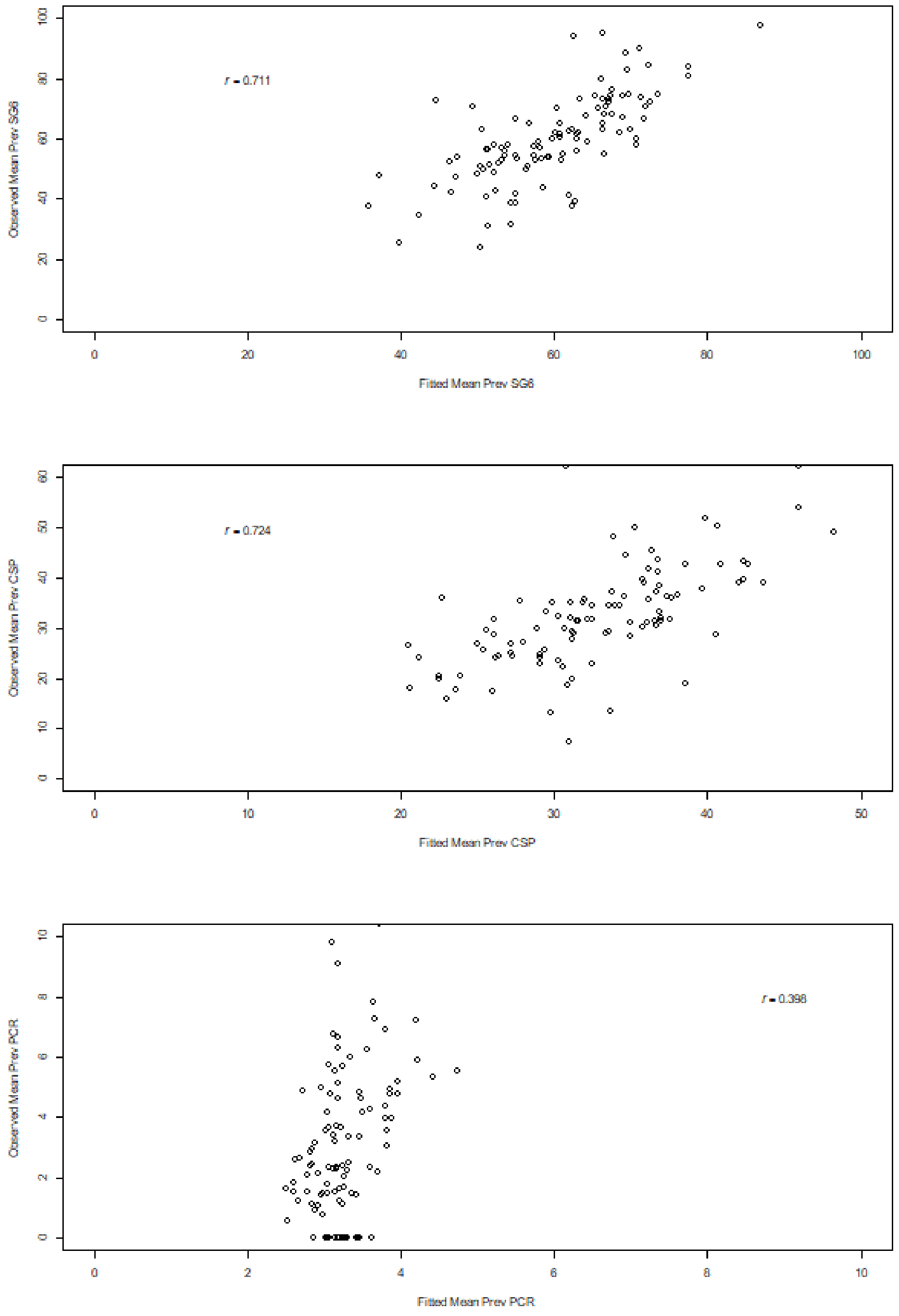

Fig. S8. Joint model of SG6, CSP and PCR - goodness of fit. Observed vs model fitted prevalence of each outcome in each village: SG6 IgG, CSP and PCR. Pearson correlation used to estimate r.

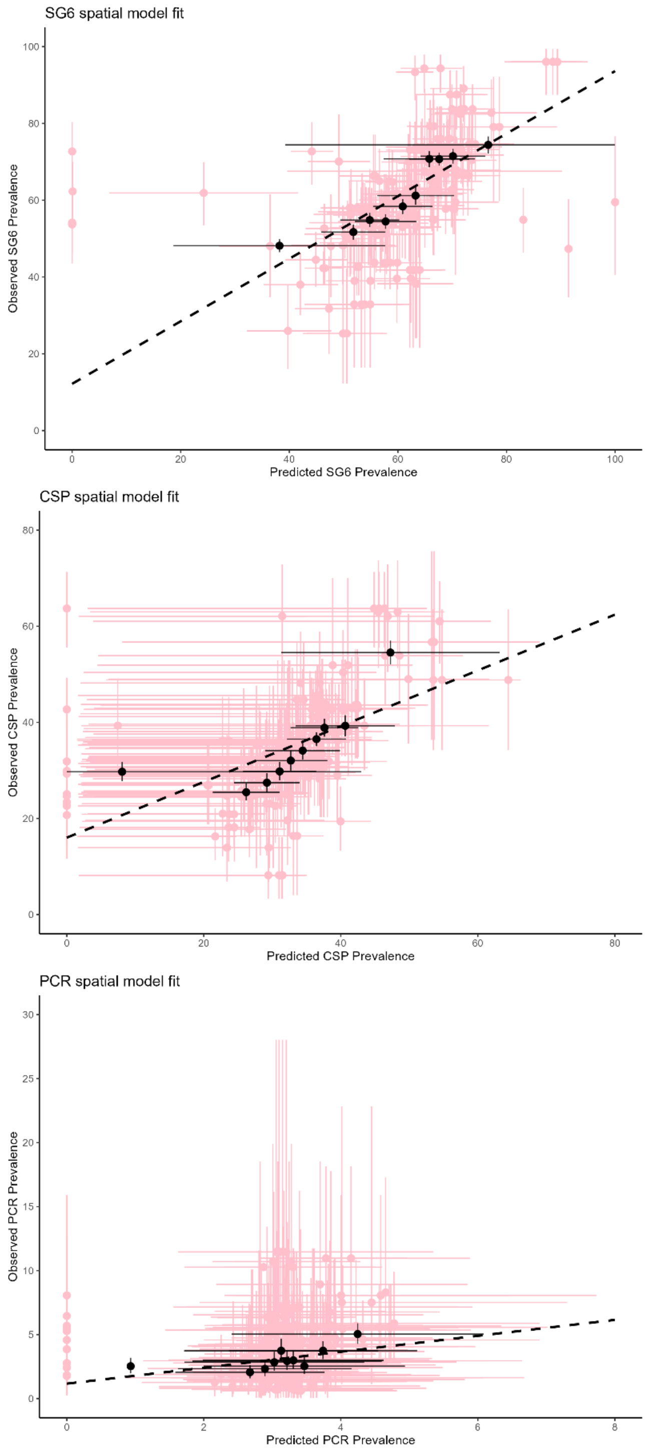

Fig. S9. Joint model validation procedure 1. The model is trained using the observed SG6, CSP and *Plasmodium* prevalence data from 90% of villages. The predicted and observed seroprevalence (with 95% credible intervals) are given for each of the omitted 10% of villages (20 repeats) represented by pink crosses and for omitted sites grouped in a series of bins (deciles) by predicted seroprevalence (black dots). Pearson correlation used to estimate: SG6 r = 0.926, CSP r = 0.723 and *Plasmodium* prevalence r = 0.615

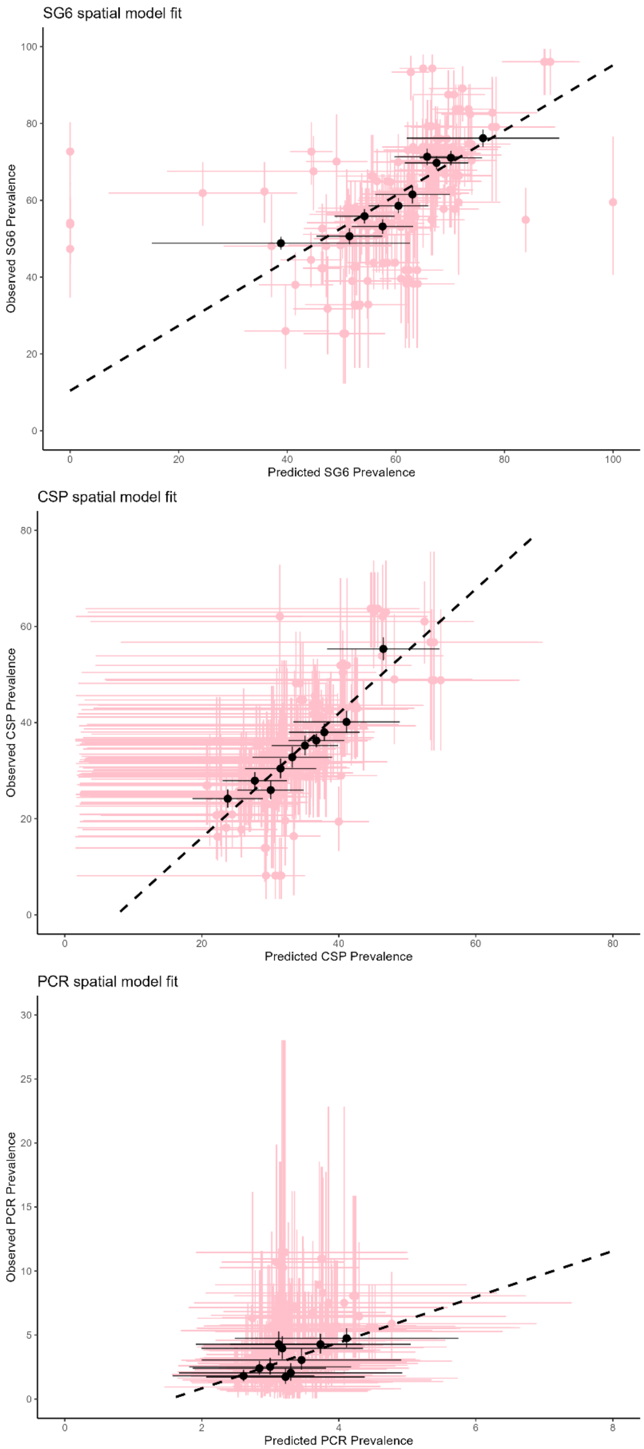

Fig. S10. Joint model validation procedure 2. The model is trained using the observed SG6 data from 90% of villages, and the CSP and PCR data from all villages. The predicted and observed seroprevalence (with 95% credible intervals) are given for each of the omitted 10% of villages (20 repeats) represented by pink crosses and for omitted sites grouped in a series of bins (deciles) by predicted seroprevalence (black dots). Pearson correlation used to estimate: SG6 r = 0.923, CSP r = 0.955 and *Plasmodium* prevalence r = 0.679

Supporting Information Discussion 3

Model validation procedures for joint modelling of SG6, *Pf*- and *Pv*-CSP and PCR-detectable *P. falciparum* and *P. vivax* prevalence.

Given innate differences in the biology of *Plasmodium falciparum* and *P. vivax* malaria as they relate to transmission and the establishment of infection (*i.e. P. vivax* infections can be caused from relapsing hypnozoites rather than new infectious bites), here we present the novel joint modelling approach for each species. Fig. S11 shows the predicted seroprevalence of antibodies against the *Anopheles* salivary antigen SG6 and *P. falciparum* sporozoite antigen *Pf*CSP, as well as the prevalence of PCR-detectable *P. falciparum* infections. While Fig. S14 indicates the predicted seroprevalence of anti-SG6 and anti-*Pv*CSP IgG antibodies, as well as the predicted prevalence of *P. vivax* infections across the region of Southeast Myanmar. The foci of transmission identified are similar between joint models. Table S5 and Table S6 indicates the regression coefficients for variables identified using respective stepwise model selection procedures for *P. falciparum* and *P. vivax* transmission. Fig. S12 and Fig. S15 indicate the recovery of positive associations between each of outcomes after joint modelling and Fig. S13 and Fig. S16 show good predictive power of these joint models.

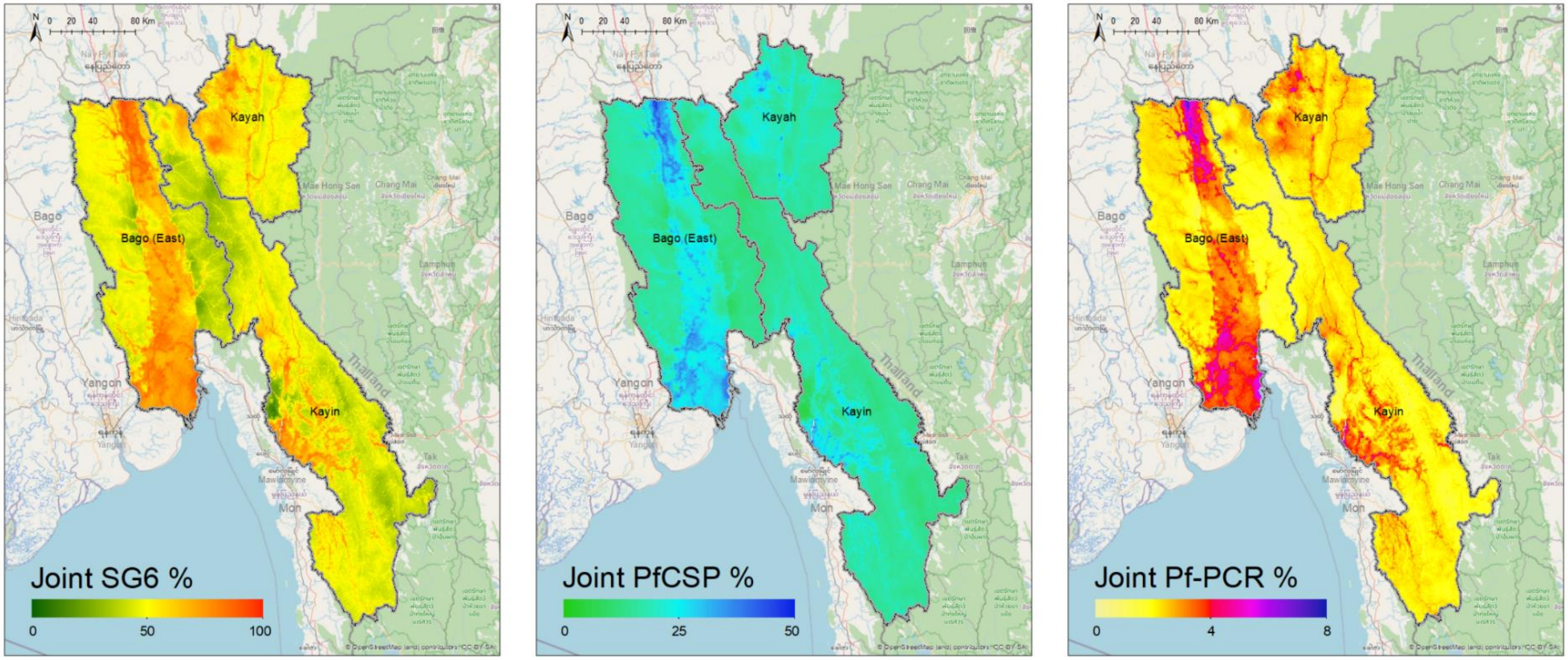

Fig. S11. Predicted seroprevalence of anti-SG6 and *Pf*CSP IgG antibodies and predicted prevalence of PCR-detectable *P. falciparum* infections, after joint modelling. Estimated using a geospatial model that adjusts for land surface temperature (diurnal difference), potential evapotranspiration, topographical wetness index, distance to water, slope, tree coverage fraction and night-time lights.

Table S5. Regression coefficients from joint model of SG6, *Pf*CSP and *Pf*-PCR prevalence.

	**OR**	**95% CrI**	
Intercept SG6	1.26	0.75	2.12
Intercept *Pf*CSP	0.19	0.17	0.21
Intercept *Pf*-PCR	0.04	0.03	0.05
Slope SG6	1.66	1.30	2.11
Slope *Pf*CSP	1.23	0.86	1.77
Slope *Pf*-PCR	1.49	0.86	2.57
Land Surface Temperature (Diurnal Difference)	0.78	0.63	0.98
Potential Evapotranspiration	1.14	0.92	1.41
Distance to water	0.84	0.69	1.02
Topographical wetness index	1.07	0.93	1.23
Slope	0.90	0.82	0.99
Tree coverage fraction	0.58	0.43	0.78
Night-time lights	1.17	1.01	1.34

Note. Table shows the odds ratio (OR) and 95% credible interval (95%CrI) from a Bayesian geostatistical joint model with multiple likelihoods. Models were fitted to participant data from all villages who had observations for each outcome (n=11,988), with each model adjusting for covariates: land surface temperature (diurnal difference), potential evapotranspiration, topographical wetness index, distance to water, slope, tree coverage fraction and night-time lights (scaled for a 1 standard deviation change).

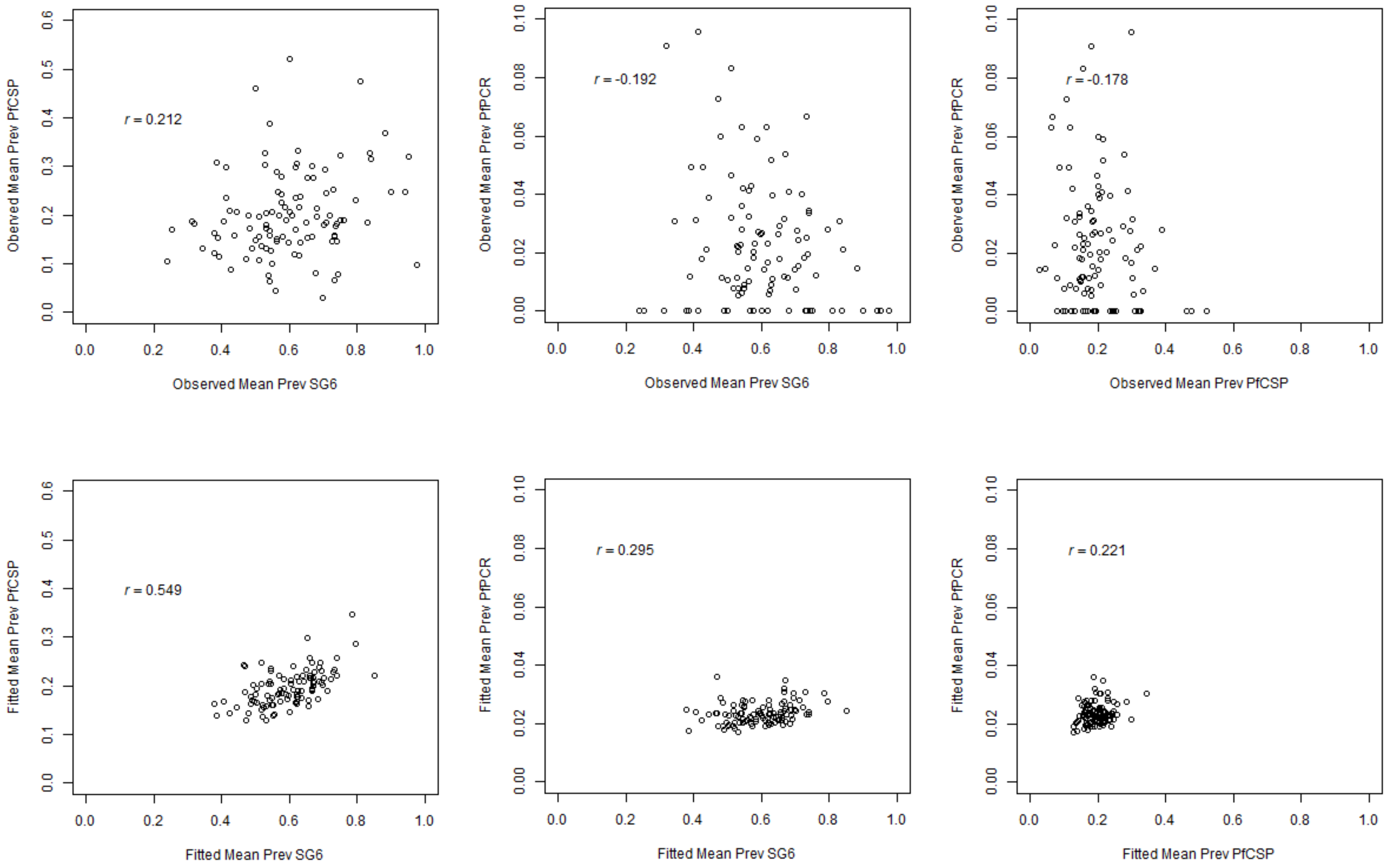

Fig. S12. Associations between outcomes of interest (SG6, *Pf*CSP, *Pf*-PCR) in each village, both before (observed) and after (fitted) joint modelling. Pearson correlation used to estimate r.

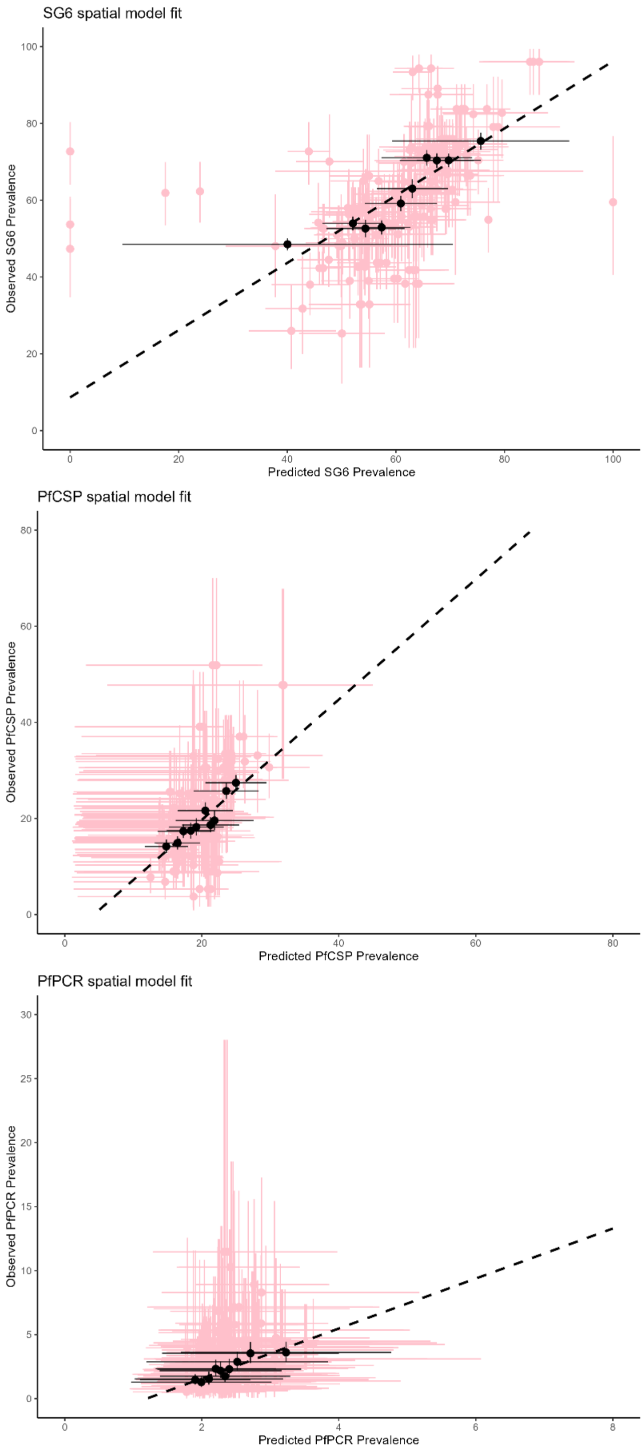

Fig. S13. Model validation procedure. The model is trained using the observed SG6 data from 90% of villages, and the *Pf*CSP and *P. falciparum* PCR data from all villages. The predicted and observed seroprevalence (with 95% credible intervals) are given for each of the omitted 10% of villages (20 repeats) represented by pink crosses and for omitted sites grouped in a series of bins (deciles) by predicted seroprevalence (black dots). Pearson correlation used to estimate: SG6: r = 0.930, *Pf*CSP: r = 0.932, *Pf*-PCR: r = 0.910.

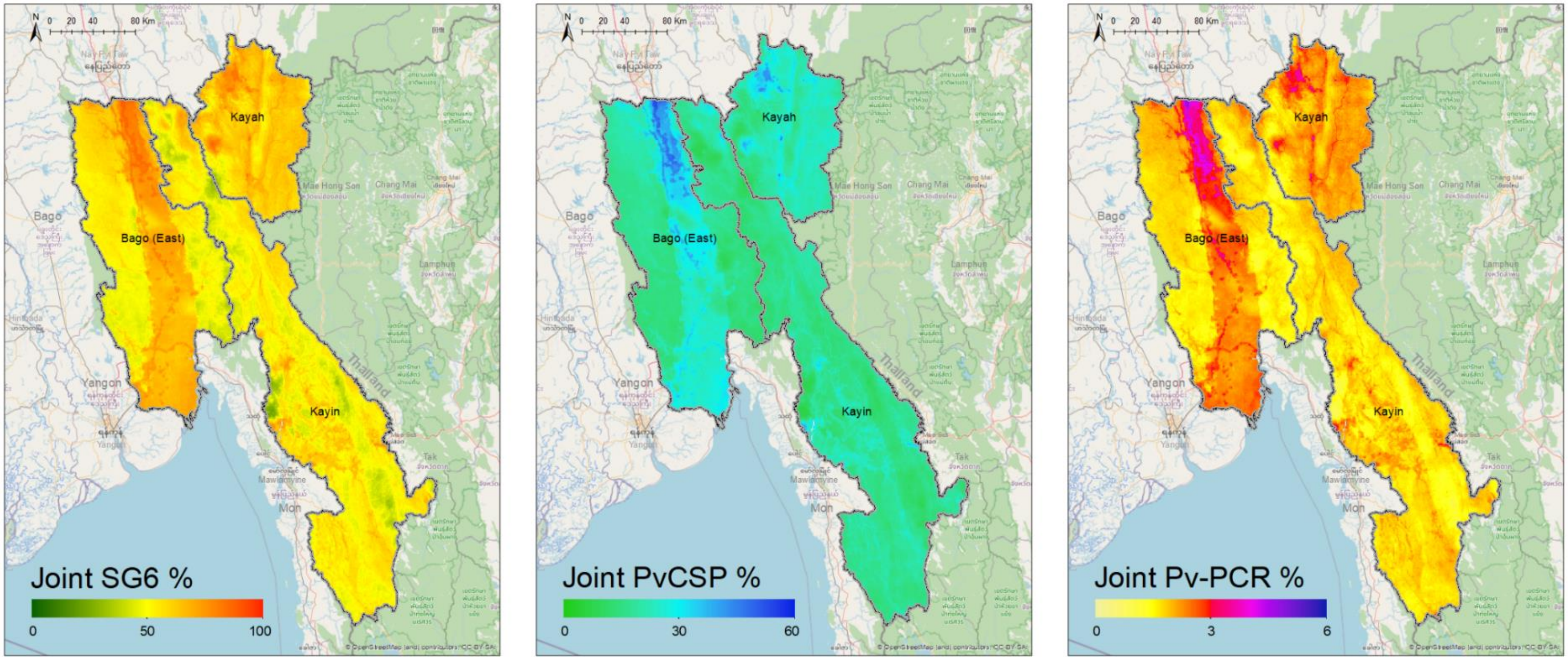

Fig. S14. Predicted seroprevalence of SG6 and *Pv*CSP IgG antibodies and predicted prevalence of PCR-detectable *P. vivax* infections, after joint modelling. Estimated using a geospatial model that adjusts for rainfall, land surface temperature (diurnal difference), potential evapotranspiration, distance to water, slope and tree coverage fraction, accessibility to cities and night-time lights.

Table S6. Regression coefficients from joint model of SG6, *Pv*CSP and *Pv*-PCR prevalence.

	**OR**	**95% CrI**	
Intercept SG6	1.42	1.09	1.85
Intercept *Pv*CSP	0.16	0.14	0.19
Intercept *Pv*-PCR	0.03	0.03	0.04
Slope SG6	2.35	1.73	3.18
Slope *Pv*CSP	1.15	0.82	1.61
Slope *Pv*-PCR	1.49	1.04	2.15
Rainfall	0.82	0.65	1.04
Land Surface Temperature (Diurnal Difference)	0.96	0.79	1.17
Potential Evapotranspiration	1.22	1.02	1.46
Distance to water	0.97	0.81	1.15
Slope	0.88	0.82	0.96
Tree coverage fraction	0.77	0.61	0.98
Accessibility to cities	1.08	0.93	1.25
Night-time lights	1.12	1.00	1.26

Note. Table shows the odds ratio (OR) and 95% credible interval (95%CrI) from a Bayesian geostatistical joint model with multiple likelihoods. Models were fitted to participant data who had observations for each outcome (n=11,988), with each model adjusting for covariates: rainfall, land surface temperature (diurnal difference), potential evapotranspiration, distance to water, slope and tree coverage fraction, accessibility to cities and night-time lights (scaled for a 1 standard deviation change).

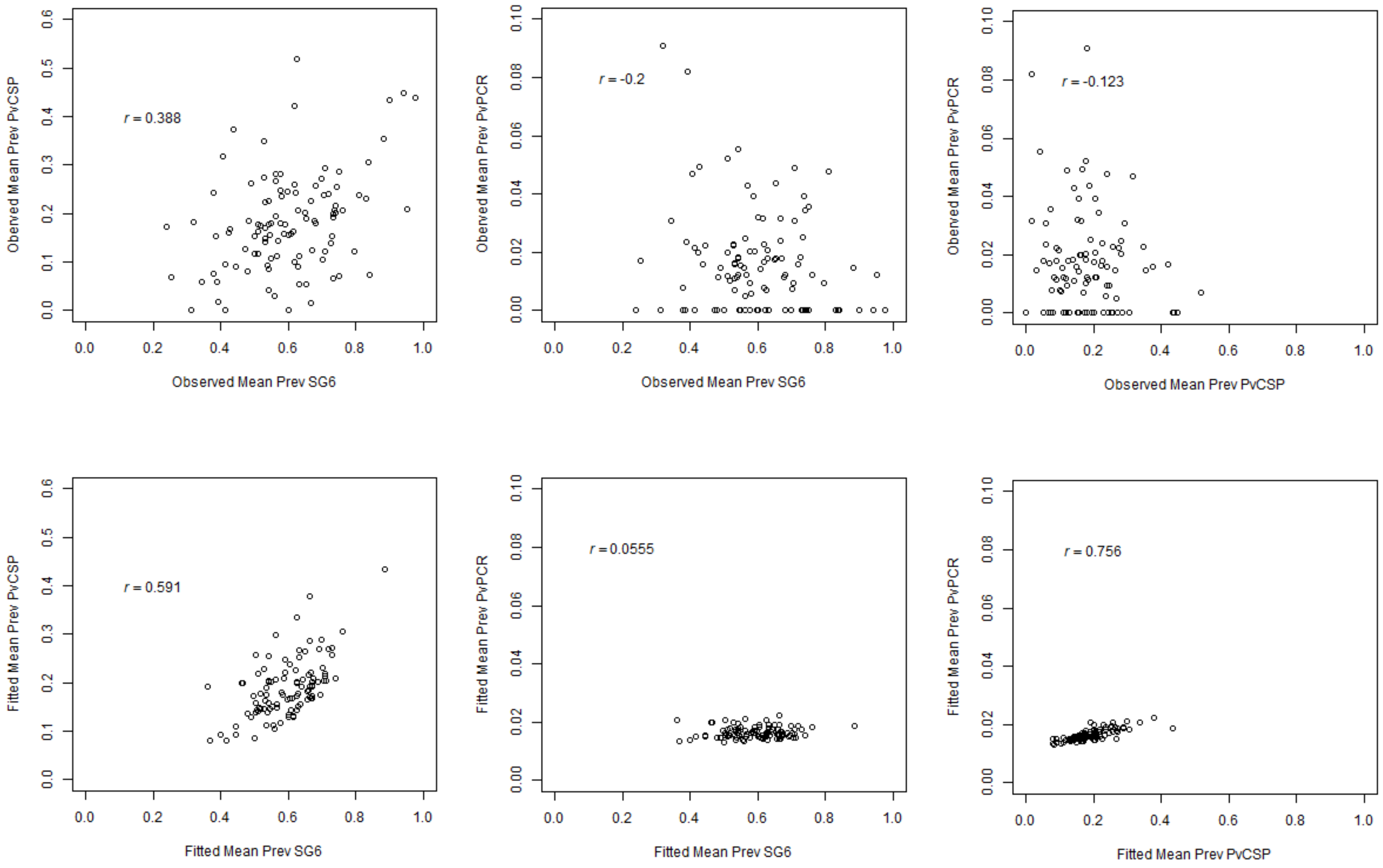

Fig. S15. Associations between outcomes of interest (SG6, *Pv*CSP, *Pv*-PCR) in each village, both before (observed) and after (fitted) joint modelling. Pearson correlation used to estimate r.

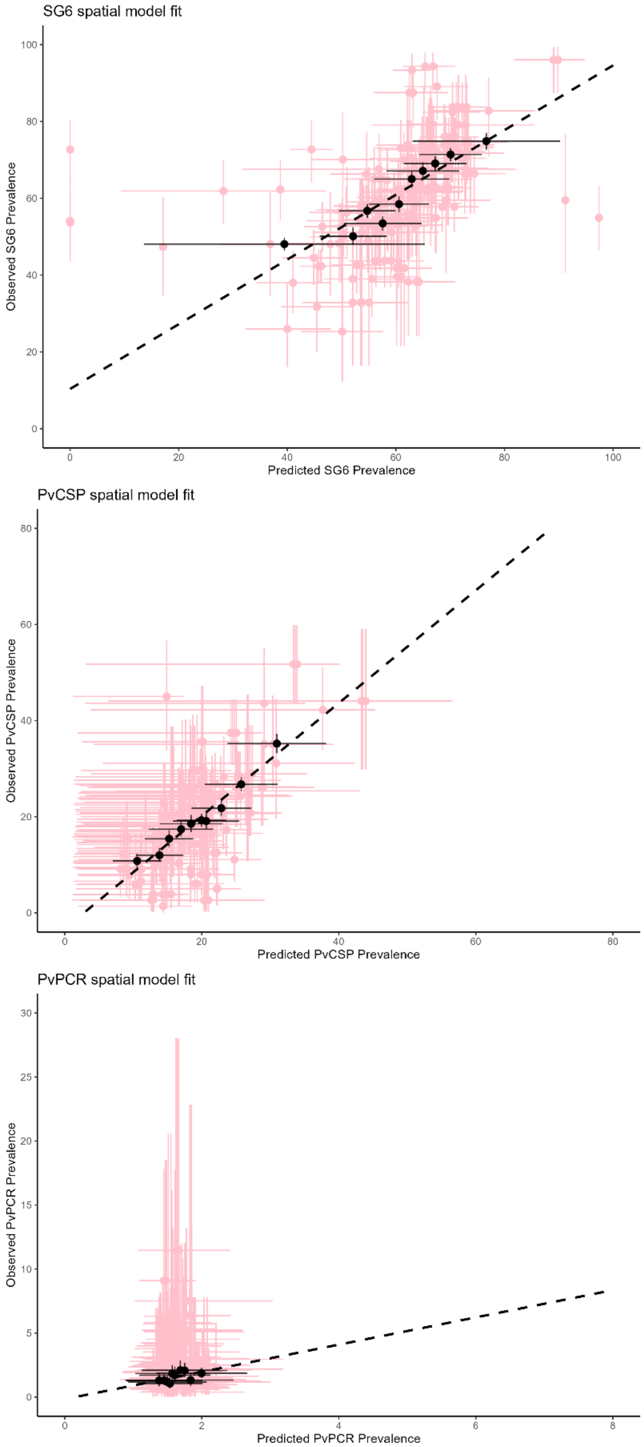

Fig. S16. Model validation procedure. The model is trained using the observed SG6 data from 90% of villages, and all of the *Pv*CSP and *P. vivax* PCR prevalence data. The predicted and observed seroprevalence (with 95% credible intervals) are given for each of the omitted 10% of villages (20 repeats) represented by pink crosses and for omitted sites grouped in a series of bins (deciles) by predicted seroprevalence (black dots). Pearson correlation used to estimate: SG6: r = 0.919, *Pv*CSP: r = 0.982, *Pv*-PCR: r = 0.614.

Supporting Information Methods

Study location

This study uses data from a stepped-wedge cluster randomised controlled trial assessing the effectiveness of personalised insect repellent delivered by village health volunteers performing routine malaria services. The study was performed in 114 hard to reach villages from three states (Kayin, Kayah and Bago (East)) in Southeast Myanmar. Geolocations (longitude and latitude) of villages were determined retrospectively using Place codes (Pcodes) from the Myanmar Information Management Unit (MIMU) database which links Pcodes with GPS coordinates. In instances where a villages' Pcode could not be matched or MIMU did not provide GPS coordinates for a Pcode (35 villages), we arranged with the original conductors of the study to revisit villages and were able to retrieve GPS coordinates for an additional 29 villages. In total, data were collected for 104 villages (Fig. S17).

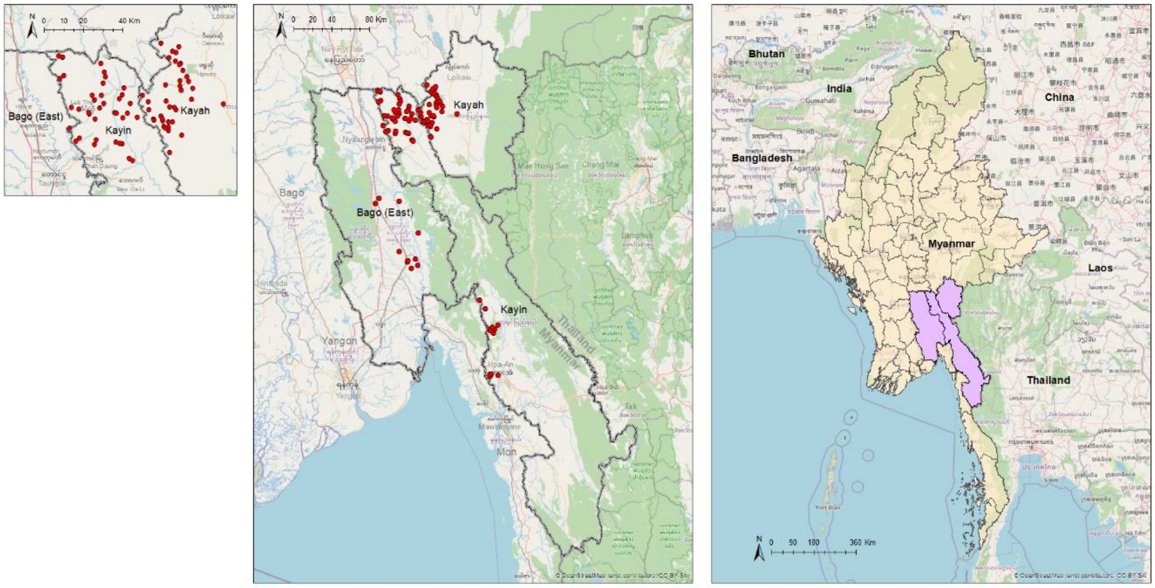

Fig. S17. Location of 104 study villages from Kayin, Kayah and Bago (East) states in Southeast Myanmar. Map was generated in ArcMap using shapefiles from Malaria Atlas Project and base maps from OpenStreetMap.

Anopheles salivary antibody detection.

Detection of total IgG antibodies against *Anopheles* salivary antigen gSG6 was performed by adapting previously published ELISA protocols, and optimising them into a high-throughput protocol outlined below. Spectraplates (Perkin Elmer) were coated with 0.5µg/mL of synthetic *An. gambiae* salivary peptide (gSG6-P1) (Genscript) resuspended in autoclaved MilliQ water and diluted in PBS, and incubated for 3 hours at 37ºC. Plates were washed and blocked for one hour at 37ºC with Blocking Buffer (Pierce, Thermo Scientific USA). After a subsequent wash step, sera were added at a desired concentrations (diluted in 10% Blocking Buffer with PBS) and incubated overnight at 4ºC. Following sera incubation, plates were washed and HRP-conjugated goat anti-human IgG (Millipore) was added at a 1:500 dilution. Plates were incubated at 37ºC for 1.5 hours and then washed. ABTS (2,2'-azino-bis(3-ethylbenzothiazoline-6-sulfonic acid)) substrate was added to each well, covered and left to develop at room temperature, then stopped with 1% sodium dodecyl sulphate (SDS), and the optical density (OD) was read in a spectrophotometer at 405nm.

Statistical analysis.

Validation analyses to assess the models' goodness of fit and predictive accuracy were performed by Pearson correlation of observed versus predicted data and hold out procedures, respectively. Specifically, the models were trained using observed data from a subset of 90% of the villages, and then used to predict the prevalence in the withheld villages (*i.e.* the test dataset, 10% of villages). This process was repeated 20 times with different subsets, and the observed vs predicted prevalence were compared (using Pearson correlation) for each omitted site in the test dataset and for a series of bins (deciles) by the predicted prevalence.
